# Efficient delipidation of a recombinant lung surfactant lipopeptide analogue by liquid-gel chromatography

**DOI:** 10.1371/journal.pone.0226072

**Published:** 2019-12-04

**Authors:** Oihana Basabe-Burgos, Jakub Zebialowicz Ahlström, Pavol Mikolka, Michael Landreh, Jan Johansson, Tore Curstedt, Anna Rising

**Affiliations:** 1 Department of Neurobiology, Care Sciences and Society, Division for Neurogeriatrics, Karolinska Institutet, Huddinge, Sweden; 2 Biomedical Center Martin and Department of Physiology, Jessenius Faculty of Medicine in Martin, Comenius University in Bratislava, Martin, Slovakia; 3 Science for Life Laboratory, Department of Microbiology, Tumour and Cell Biology, Karolinska Institutet, Tomtebodavägen, Stockholm, Sweden; 4 Department of Molecular Medicine and Surgery, Karolinska Institutet at Karolinska University Hospital, Stockholm, Sweden; 5 Department of Anatomy, Physiology and Biochemistry, Swedish University of Agricultural Sciences, Uppsala, Sweden; Helsingin Yliopisto, FINLAND

## Abstract

Pulmonary surfactant preparations extracted from natural sources have been used to treat millions of newborn babies with respiratory distress syndrome (RDS) and can possibly also be used to treat other lung diseases. Due to costly production and limited supply of animal-derived surfactants, synthetic alternatives are attractive. The water insolubility and aggregation-prone nature of the proteins present in animal-derived surfactant preparations have complicated development of artificial surfactant. A non-aggregating analog of lung surfactant protein C, SP-C33Leu is used in synthetic surfactant and we recently described an efficient method to produce rSP-C33Leu in bacteria. Here rSP-C33Leu obtained by salt precipitation of bacterial extracts was purified by two-step liquid gel chromatography and analyzed using mass spectrometry and RP-HPLC, showing that it is void of modifications and adducts. Premature New Zealand White rabbit fetuses instilled with 200mg/kg of 2% of rSP-C33Leu in phospholipids and ventilated with a positive end expiratory pressure showed increased tidal volumes and lung gas volumes compared to animals treated with phospholipids only. This shows that rSP-C33Leu can be purified from bacterial lipids and that rSP-C33Leu surfactant is active against experimental RDS.

## Introduction

Pulmonary surfactant lines the terminal air spaces and reduces the surface tension of the alveolar liquid layer, which is necessary for pulmonary compliance and alveolar patency at end-expiration [1]. The main component of lung surfactant is phospholipids. Phosphatidylcholines (PC) constitute 60–70% of surfactant phospholipids and anionic phospholipids, mainly phosphatidylglycerol (PG) constitutes 8–15%. Unlike other biological membranes, lung surfactant contains a very high percentage of 1,2-dipalmitoyl-*sn*-glycero-3-phosphocholine (DPPC) [2]. In addition to the phospholipids, there are four surfactant-associated proteins (SP): SP-A, SP-B, SP-C and SP-D. SP-B and SP-C are hydrophobic and mediate adsorption of phospholipids to the air-water interface, which reduces the surface tension [3–5]. SP-A and SP-D, on the other hand, are large water soluble collectins that primarily are involved in innate immunity [6].

Deficiency of endogenous pulmonary surfactant in premature children leads to neonatal respiratory distress syndrome (RDS) that can be efficiently treated with intratracheal instillation of animal-derived surfactant preparations containing phospholipids and the hydrophobic proteins SP-B and SP-C [7]. The properties of exogenous surfactant preparations, in particular their ability to spread to distal parts of the lungs, make them attractive also for development of novel pulmonary drug delivery systems [8, 9] and therapies against acute RDS (ARDS) [10, 11]. However, high cost and limited supply of animal-derived surfactant preparations hamper the development of such applications [12].

The development of synthetic lung surfactants have been challenging, mainly due to difficulties in producing SP-C and SP-B and analogues thereof [12]. SP-B is a peripheral membrane protein with a complex tertiary structure containing three intra- and one intermolecular disulfide bridges, giving rise to a covalent homodimer [13]. SP-C is a transmembrane lipopeptide that contains a single hydrophobic alpha-helix made of a poly-valine stretch, as well as two palmitoylated cysteines at positions 5 and 6 [14, 15]. The SP-C helix spans a phospholipid bilayer and may also be present in monolayers in a tilted position [16]. The poly-valine stretch of SP-C is inherently prone to form beta-sheets and may aggregate into amyloid-like fibrils [17]. To circumvent this problem SP-C33Leu was designed in which the poly-valine region is replaced by a poly-leucine region, but with one Leu in the N-terminal part of the alpha-helix substituted with one Lys, two Cys are replaced with Ser and two N-terminal residues are truncated [18–21]. The analogue SP-C33Leu and its palmitoylated counterpart can be produced by chemical synthesis [22] and is the main protein constituent of the artificial surfactant preparation CHF5633 [23]. CHF5633 has shown safety and efficacy in a phase I clinical trial in infants with RDS [24].

Our group has recently shown that transmembrane and other aggregation-prone proteins can be efficiently produced in a heterologous system using a modified version of an N-terminal domain of a spider silk protein (NT*) as a solubility tag [25, 26]. Recombinant SP-C33Leu (rSP-C33Leu) produced in this way and purified by a simple salt precipitation and organic solvent extraction method was effective in increasing compliance and lung gas volumes compared to non-treated littermates in a premature rabbit fetus model of RDS [25]. Although this method of producing rSP-C33Leu is cost-efficient and removes protein impurities, it does not efficiently remove bacterial lipid contaminants. The aims of this study were to establish an efficient method for delipidation of rSP-C33Leu, and to test rSP-C33Leu-based lung surfactant in a rabbit model of neonatal RDS.

## Materials and methods

### Expression, NaCl precipitation, cleavage of NT*-SP-C33Leu and extraction of rSP-C33Leu

A DNA construct coding for a His_6_-tag, the NT* solubility tag, a strategically placed methionine that allows cyanogen bromide (CNBr) cleavage and the target peptide SP-C33Leu was cloned into pT7 vector, transformed into *Escherichia coli* BL21 and expressed as described by Kronqvist et al [25]. The cells were lysed using a cell disrupter (T‐S Series Machine, Constant Systems Limited, Daventry, Northants, UK) at 30 kPa and soluble and insoluble fractions were separated by centrifugation at 24,000 x g, at 4°C for 30 min. Sodium chloride was added to the supernatant fraction containing NT*-SP-C33Leu to a final concentration of 1.2 M and it was then centrifuged at 50,000 x g for 30min, the supernatant obtained was discarded, and the pellet containing NT*-SP-C33Leu was dissolved in 20 mM Tris, pH 8 and sonicated at 60% amplitude, 1s on and 1s off for 3 min. Cleavage was performed overnight at room temperature by adding 50 mM CNBr and 100mM HCl to reduce pH to 1. Then, 400 mM NaCl was added to the cleavage reaction in order to precipitate rSP-C33Leu, followed by centrifugation at 15,000 x g for 30 min. The supernatant was discarded and the pellet was redissolved in methanol:dichloroethane:H_2_O 85:10:5 (v/v/v). Insoluble material was removed by centrifugation at 50,000 x g for 30 min. The presence of the protein in each step was observed by SDS polyacrylamide electrophoresis (SDS-PAGE) using 4–20% acrylamide gradient gels (Bio-Rad Laboratories, Hercules, CA) stained with Coomassie Brilliant Blue.

### Purification of rSP-C33Leu by two-step liquid gel chromatography

The crude extract in the methanol:dichloroethane:H_2_O 85:10:5 (by vol) phase was dried under reduced pressure at 37°C and then resuspended in a 8–10 ml, corresponding to 20–25% of the column volume, of the same solvent mixture. If the solution was not clear the sample was centrifuged at 200 x g for 5 min to remove non-dissolved NaCl and the supernatant was loaded on a 2.5 cm x 8 cm Lipidex-5000 (PerkinElmer, Waltham, MA, USA) column equilibrated in methanol:dichloroethane:H_2_O 85:10:5 (by vol). Fractions were collected, dried under reduced pressure at 37°C, and weighed. The first fraction corresponding to 0.7–1 column volume, containing rSP-C33Leu as well as polar lipids, was dried, redissolved in about 4 ml of methanol:dichloroethane:H_2_O 85:10:5 (by vol), corresponding to 1% of the column volume and centrifuged at 70 x g for 5 min to further remove NaCl. The supernatant was loaded on a 2.5 cm x 80 cm Lipidex-5000 column in methanol:dichloroethane:H_2_O 85:10:5 (by vol). One fraction of 100 ml followed by fractions of 10 ml were collected, dried under reduced pressure and weighed. An aliquot of each fraction was analyzed by SDS-PAGE as described above. Fractions that contained rSP-C33Leu were dried and stored at -20°C.

### Mass spectrometry

Mass spectra were recorded on an Orbitrap Fusion (Thermo Fisher Scientific, Waltham, MA) equipped with an offline nanoelectrospray source. Samples were introduced using gold-coated Proxeon borosilicate capillaries (Thermo Scientific, Waltham, MA). The instrument was operated in positive ion and standard pressure mode. The capillary voltage was 1.4 kV, the transfer tube temperature was maintained at 40°C and the pressure in the ion-routing multipole was 0.008 torr. Spectra were recorded using the Orbitrap mass analyzer at a resolution of 120,000 at *m/z* 200 with a standard mass range acquisition window of 600–2,000 *m/z* and an AGC target of 1,000,000. Data were analyzed using the Xcalibur 3.0 software package (Thermo Scientichfic, Waltham, MA).

### Reverse-phase high performance liquid chromatography (RP-HPLC)

RP-HPLC was performed according to Gustafsson et al [27] using an Äkta pure system with a Kromasil 100–5 C18 column. The column was equilibrated in 40% aqueous ethanol (v/v) with 0.1% trifluoroacetic acid (TFA) (v/v). Samples of relevant fractions after Lipidex chromatography were dried under nitrogen stream, and resuspended in 500uL of 0.1% TFA in ethanol before injection into the column. For elution, the concentration of 2-propanol was gradually increased from 0% to 100% and absorbance at 214 nm was recorded for peptide bond detection.

### Preparation of rSP-C33Leu surfactant

DPPC and 1-palmitoyl-2-oleoyl-*sn*-glycero-3-phosphoglycerol (POPG) (both 99% purity) were from Sigma-Aldrich, St. Louis, Missouri, USA. Egg yolk-PC (95% purity) was purchased from Avanti Polar Lipids. DPPC:egg yolk-PC:POPG in 50:40:10 weight ratio were dissolved in chloroform:methanol 2:1 (v/v). rSP-C33Leu was dissolved in chloroform:methanol 1:1 (v/v). Phospholipids and rSP-C33Leu were combined in a 100:2 weight ratio and the mixture was dried under reduced pressure and resuspended in 0.9% NaCl to 80 mg/ml of phospholipids by bath sonication and repeated freeze-thaw cycles. The rSP-C33Leu surfactant was stored at -20°C. We chose to use 2% rSP-C33Leu based on that the natural derived surfactant preparation poractant alfa contains around 1.5% surfactant proteins [28] and increased concentrations (e.g 4% SP-C33Leu) does not improve the performance of synthetic lung surfactant preparations [29].

### *In vivo* experiments

Pregnant wild-type New Zealand White rabbits were kept in separate boxes in the animal facility. The animals had access to nesting material and a dark separate part of the cage for nesting, and were fed hay, water and pellets ad libum as well as fresh vegetables and fruit daily. The animals also have sticks for gnawing according to Karolinska Institutets animal enrichment plan. Health checks were made daily and the animals were assessed as healthy (score <0.3 according to Karolinska Institutets health assessment scale). On the day of the experiment, the animals were given sedatives (diazepam), and were subsequently anestethized with medetomidine and ketaminol, and were taken to the laboratorium. During surgery, the animals were given oxygen and sufficient depth of anesthesia was ensured by pinching the rabbit’s paw and testing corneal reflex. When all pups had been delivered the adult rabbit was immediately euthanized. The premature pups (of both genders) were delivered by caesarian section at 27 gestational days (term 31 days) and weighted. The pups were anaesthetized with ketamine 20 mg/kg and medetomidine 0.1 mg/kg intraperitoneally. Tracheotomy was performed and a canula was inserted in the trachea. The pups received via tracheal instillation one of four treatments assigned by a rotating scheme: 1, DPPC:Egg-PC:POPG 50:40:10 200 mg/kg (80mg/ml) (n = 40); 2, DPPC:Egg-PC:POPG 50:40:10 with 2% rSP-C33Leu 200 mg/kg (80mg/ml) (n = 21); 3, poractant alfa 200 mg/kg (80mg/ml) (n = 36); or 4, no treatment (n = 37), and were put in pletysmography boxes at 37°C connected in parallel to pressure-constant ventilator system (Servo Ventilator 900 B; Siemens-Elema, Solna, Sweden). Other methods for randomization are not recommended in these types of experiment since the maturity of the fetuses in litters can vary and the number of fetuses is not known until the last pup is delivered. The weight of the fetuses was 28 ± 4 g and there were no weight differences between the groups. Out of the 134 animals included in the experiment, 4 were excluded of the analysis because they got pneumothorax in the first 30 min of ventilation, 7 because the controls had tidal volumes higher than 5.5 ml/kg at 5min of ventilation and 2 because of technical problems. In total, six animals were excluded from group 1, three from group 2, two from group 3 and four from group 4. The group size recommended for this type of experiments is >8 [25, 30].

Lungs were opened with peak inspiratory pressure (PIP) of 35 cmH_2_O and zero end-expiratory pressure (PEEP) for 1 min. The rabbits were then ventilated for 35 min according to the scheme: 15 min at PIP 23 cmH_2_O/PEEP 3 cmH_2_O, 5 min at PIP 18 cmH_2_O /PEEP 3 cmH_2_O, 5 min at PIP 13 cmH_2_O/PEEP 3 cmH_2_O, 5 min at PIP 23 cmH_2_O/PEEP 3 cmH_2_O and finally 5 min with 100% N_2_ at PIP 23cmH_2_O and PEEP 3 cmH_2_O. During the entire period pups were ventilated with respiratory rate 40 breaths/min, inspiration:expiration ratio 1:1 and O_2_ 21% (except for the final 5 min) and tidal volumes were recorded every five minutes. The animals were sacrificed with pentobarbital, the lungs were excised and photographed, and the lung gas volumes were measured by water displacement method [31]. T-test was used to analyze the lung gas volumes and the tidal volumes were analyzed using two-way anova. The statistical analysis was done using GraphPad software (Graph Pad Software, Inc., San Diego, CA). The experiments were performed during day-time and in the laboratory. No adverse events occurred.

### Ethical permit

The animal experiments followed the ethical permit (N174/14) approved by Stockholms Norra Djurförsöksetiska Nämnd.

## Results

### Purification of rSP-C33Leu by liquid gel chromatography in organic solvents

**Fig 1:**
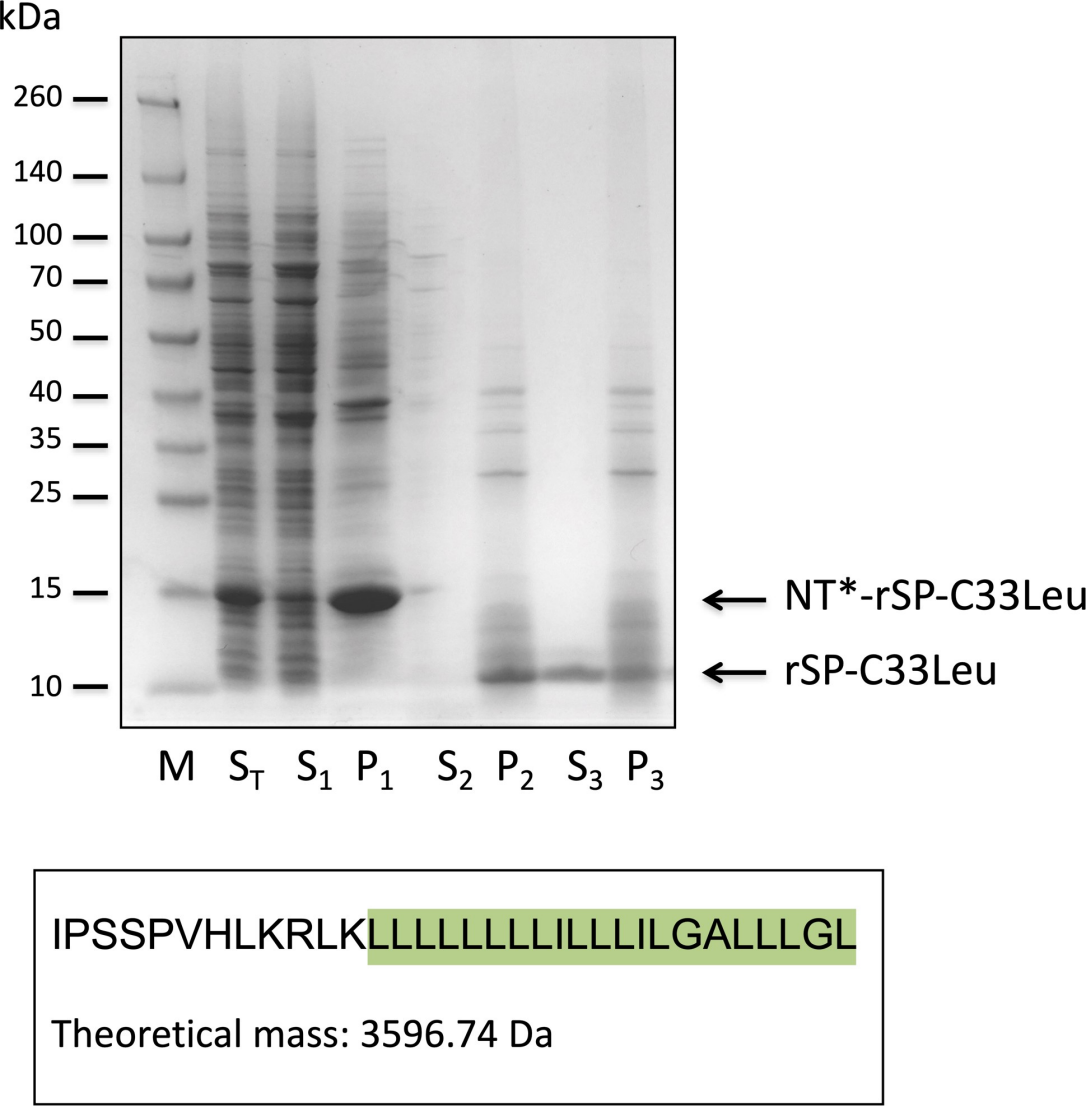
Non-chromatographic purification of rSP-C33Leu. SDS-PAGE indicating the different purification steps of rSP-C33Leu. The lanes show supernatant (S_T_) after cell disruption, supernatant (S_1_) and pellet (P_1_) after NaCl precipitation, supernatant (S_2_) and pellet (P_2_) after CNBr cleavage and supernatant (S_3_) and pellet (P_3_) after resuspension in methanol:dicloroethane:H_2_O 85:10:5 (by vol). The lane labeled M shows migration of molecular size markers with identified masses i kDa. The amino acid sequence and the theoretical average molecular mass of rSP-C33Leu are shown below the SDS-PAGE, and the residues corresponding to the transmembrane region are shaded.

**Fig 2:**
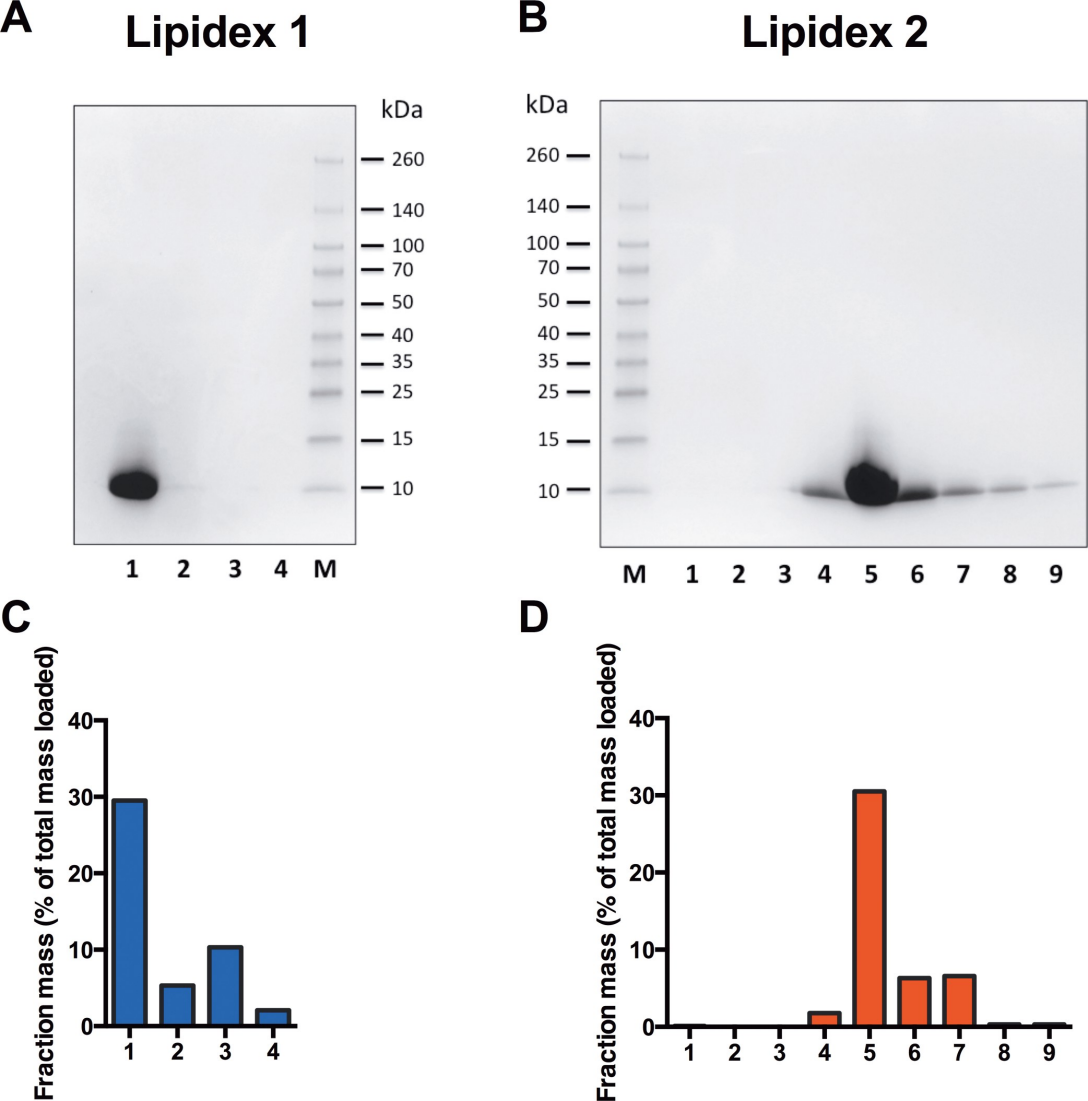
Liquid gel chromatography of organic extract. The organic extract containing rSP-C33Leu and obtained by salt precipitation and resolubilisation was purified by two consecutive Lipidex-5000 columns. SDS-PAGE and dry weight % of different fractions in relation to the total amount put on the column was determined for the 8 cm long first column (A and C), and the second 80 cm long column (B and D). In the first column (C), fraction 1 corresponds to a volume of 28 ml (0.7 column volume), fraction 2 to 20 ml (0.5 column volumes) and fractions 3 and 4 to 40 ml (1 column volume) each. In the second column (D), fraction 1 corresponds to a volume of 100 ml (0.25 column volumes) and the rest of the fractions to 10 ml (0.025 column volumes) each.

**Fig 3:**
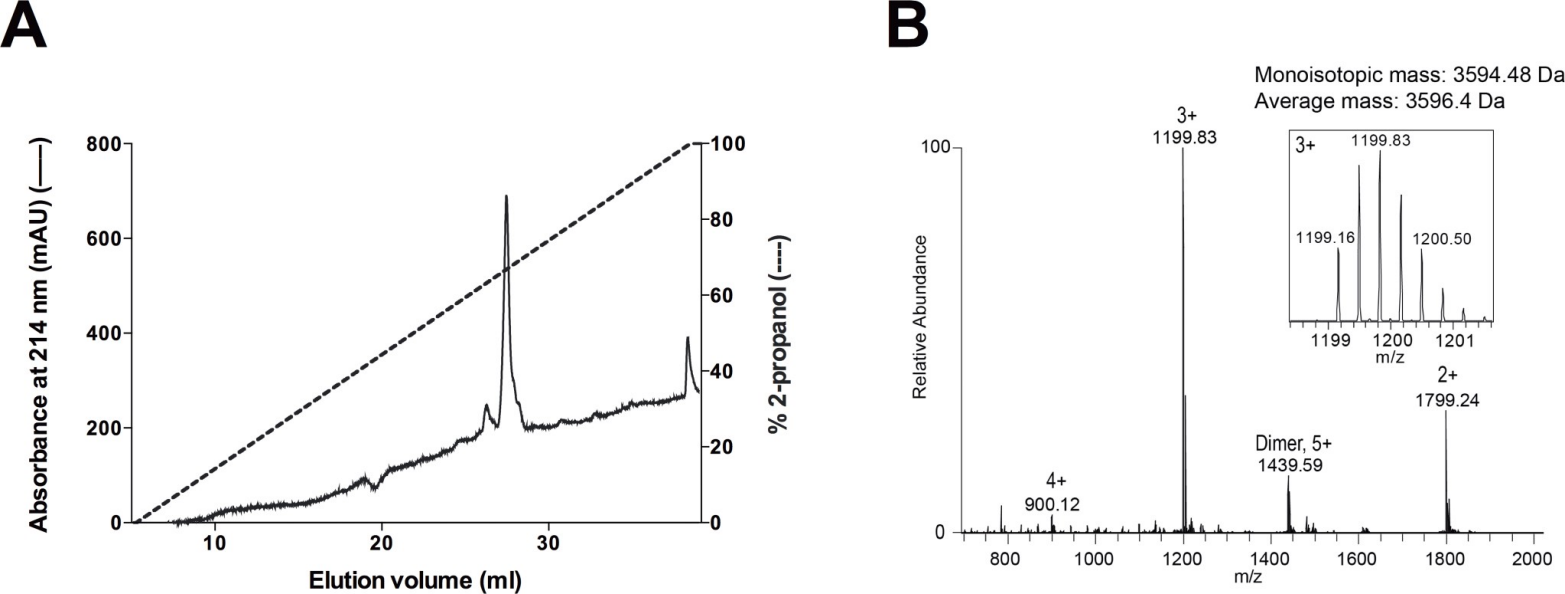
High-performance liquid chromatography and mass spectrometry of Lipidex-5000 purified rSP-C33Leu. The peptide obtained from the second Lipidex-5000 column (Fig 2B and 2D, fractions 4 and 5) was analyzed by RP-HPLC (A) and mass spectrometry (B). The close-up on the 3+ charge state show 0.33m/z intervals.

The non-chromatographic purification procedure previously described [25] efficiently purifies NT*-SP-C33Leu and the lipophilic rSP-C33Leu from other proteins (Fig 1, lane S_3_). However, two potential problems with salt precipitation followed by solubilisation in organic solvents are that various amounts of NaCl will be found in the organic extract and that lipids of different polarity as well as many undefined components will copurify with the lipophilic peptide rSP-C33Leu. The amount of salts may be reduced by centrifugation of the samples and the first chromatographic separation was performed to further reduce the amount of NaCl and to get rid of unpolar lipids. In the first fraction, corresponding to one column volume, 57 ± 13% (mean ± SD, n = 13) of the material put on the column was eluted. By restricting the first fraction to 70% of the column volume a further purification of rSP-C33Leu was obtained. This fraction then contained 34 ± 7% (mean value ± SD, n = 4) of the original amount put on the column, and all of the rSP-C33Leu (Fig 2A and 2C). A small sample volume and a long column was a prerequisite for separation of rSP-C33Leu and polar lipids such as phospholipids. During a second separation step using a Lipidex-5000 column (2.5 x 80 cm), the majority of rSP-C33Leu eluted between 33 and 38% of one column volume, as judged by SDS-PAGE (Fig 2B and 2D). Thus, the two chromatographic steps removed NaCl, lipids and other impurities and the purified rSP-C33 fraction made up 8.2 ± 3.3% of the original material (Fig 1, lane S_3_). Material eluting in fractions corresponding to 4 and 5 in Fig 2B were used for further analyses and for surfactant preparation since most of the peptide was found in fraction 5, and the smaller size and higher hydrophobicity of lipid contaminants make them elute later from the Lipidex column. RP-HPLC was used to estimate the purity and yield of rSP-C33Leu after the two-step Lipidex-5000 column chromatography and showed one major peak that eluted at 67% 2-propanol (Fig 3A). SDS-PAGE of the main HPLC peak showed a single band corresponding to rSP-C33Leu (S1 Fig), and the presence of rSP-C33Leu was confirmed by mass spectrometry (Fig 3B). The mass spectrum showed a monoisotopic mass of rSP-C33Leu of 3594.48 Da and an average mass of 3596.4 Da, which correlates well with the theoretical average mass of 3,596.74 Da. Using chemically synthesized SP-C33Leu[23] as standard for RP-HPLC, the amount of rSP-C33Leu peptide eluting from the second Lipidex-5000 column was estimated to correspond to 70% of the dry mass. The total yield of delipidated rSP-C33Leu ranged between 5 and 10 mg per liter bacterial culture.

### *In vivo* experiments

**Fig 4:**
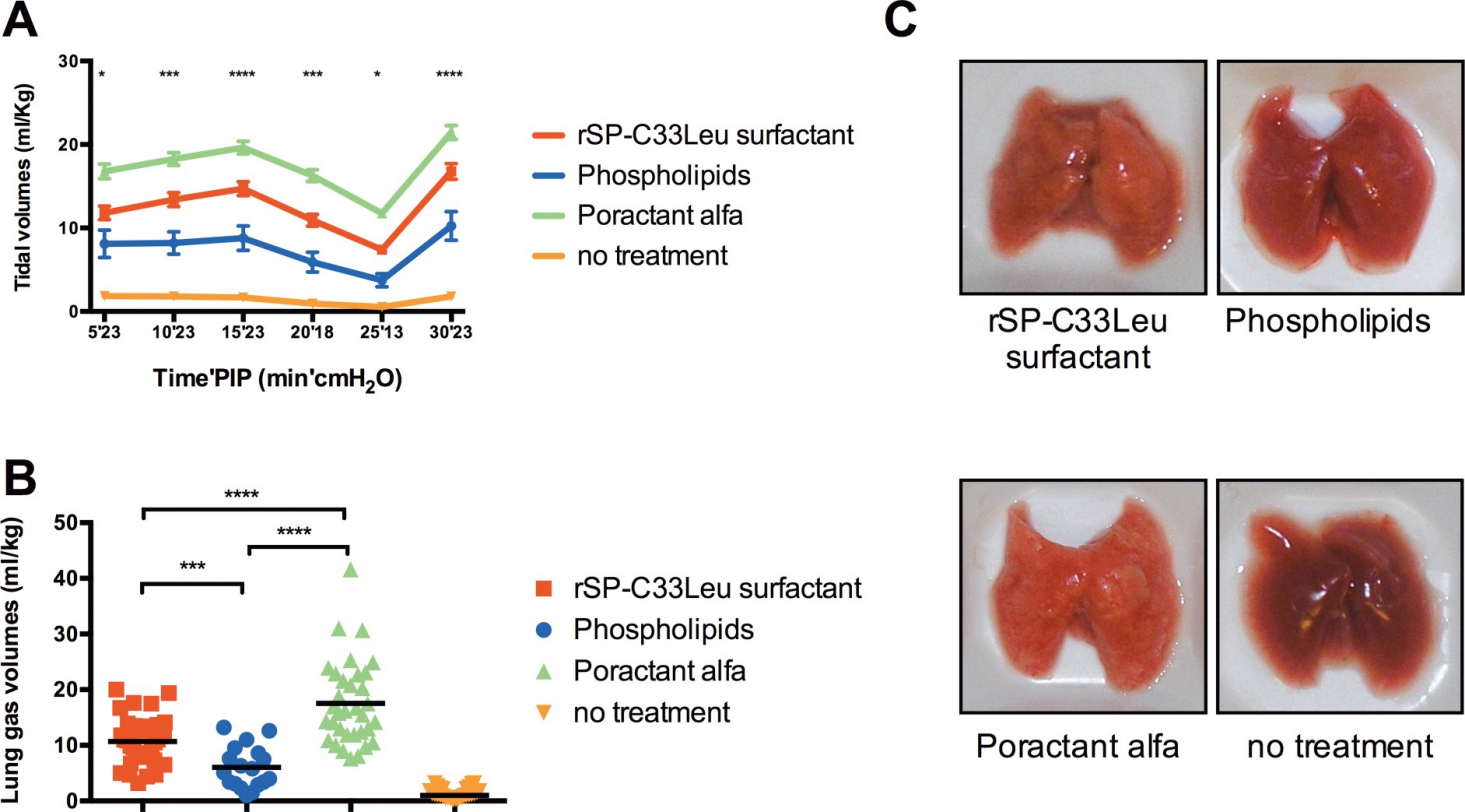
Effect of rSP-C33Leu surfactant on lung function and macroscopic appearance. (A) tidal volumes (V_T_) during 30min ventilation of premature rabbit fetuses. Mean values and standard errors are shown. p-values between rSP-C33Leu surfactant and phospholipid treated groups are given according to: * = p ≤0.05 and *** = p≤0.001. (B) lung gas volumes (LGV) measured at the end of the ventilation period. Median LGV values are indicated by horizontal lines. *** = p≤0.001 and **** = p≤0.0001. (C) representative photographs of lungs of the animals in each treatment group.

An artificial lung surfactant preparation consisting of 2% purified rSP-C33Leu (w/w) in DPPC, egg yolk-PC and POPG (50:40:10 w/w) suspended in physiological saline to 80 mg/ml was prepared. Premature rabbit pups were instilled either with the rSP-C33Leu surfactant (200mg/kg), poractant alfa (200mg/kg) or phospholipids only (200mg/kg) or were given no treatment. Tidal volumes in the poractant alfa group were significantly higher at all time points than in those for all other groups (p <0.0001) (Fig 4A). Furthermore, the rSP-C33Leu surfactant group had significantly higher the tidal volumes at all time points than the phospholipid group (p <0.02–0.0001) and all treated groups had significantly higher tidal volume than the non-treated group (p<0.0001). At the end of the experiment the lung gas volumes in the rSP-C33Leu surfactant group were higher than in the phospholipid group but lower than in the poractant alfa group (Fig 4B). The macroscopic appearances of the lungs showed more atelectatic areas in the lungs from the phospholipid treated animals than in the lungs from the rSP-C33Leu surfactant treated group (Fig 4C).

## Discussion

Although the composition of animal-derived lung surfactant preparation is highly complex, its function in replacement therapy of experimental neonatal RDS can be successfully mimicked by rather simple mixtures of phospholipids and surfactant proteins. Both SP-B and SP-C analogs are required for optimal therapeutic effect in these models when no PEEP is applied [5, 29]. The absence of SP-B can to a large extent be compensated for by stabilizing the alveoli at end expiration using ventilation with PEEP [32], which is routine in clinical treatment of RDS. The requirement of PEEP for optimal effects of surfactant preparations containing only SP-C analogs likely reflect that they are functionally inferior. However, surfactant preparations that lack SP-B analogs still spreads efficiently and lowers the surface tension, which make it possible to formulate a simple but still effective surfactant preparation from phospholipids and an analog of SP-C only.

SP-C33Leu is a strictly hydrophobic peptide, ie it is not soluble in aqueous solvents, which makes it prone to interact with bacterial phospholipids. This complicates purification, in particular when salt precipitation is employed since co-aggregates of SP-C33Leu and lipids will occur. The recently described production method of rSP-C33 [25] successfully separates the peptide from protein contaminants but does not eliminate lipids that copurify with the peptide. Although rSP-C33Leu, purified with the salt precipitation procedure only, is functionally active in animal models and also shows essentially identical activity in the captive bubble surfactometer as its synthetically produced counterpart [25, 33], the presence of lipids in rSP-C33Leu preparations is not acceptable from a regulatory point of view. In this study we describe a method to separate the peptide from lipid contaminants using liquid gel chromatography in organic solvents. The purification protocol decreased the weight obtained by the salt precipitation procedure with more than 90% and resulted in pure rSP-C33Leu with no covalent modifications as determined by RP-HPLC and mass spectrometry. By using HPLC the purity was determined to 70% (w/w), which is similar to the peptide content of native SP-B and SP-C purified by Lipidex-5000 and Sephadex LH-60 chromatography [28]. This is the first protocol that enables isolation of a recombinant SP-C analogue without the need to add detergents or lipids [34, 35], and it is likely that it is useful also for purification of other recombinant non-polar peptides.

A mixture of 2% rSP-C33Leu in 80 mg/ml phospholipids markedly increased the therapeutic effect compared to phospholipids only in a preterm rabbit model of RDS using PEEP. Lung tidal volumes and lung gas volumes were all significantly improved (Fig 4A and 4B). The macroscopic appearance of the lungs at the end of the experiment were in line with the results from the lung gas volume measurements (Fig 4B and 4C). The effect of rSP-C33Leu surfactant was lower than that of poractant alfa, which may be due to the presence of native SP-B and SP-C and a more complex phospholipid composition in poractant alfa (Fig 4). However, for e.g. drug delivery applications and for treatment of ARDS where large doses are needed and the pathophysiology is different, the rSP-C33Leu surfactant presented herein may form the basis for the development of novel treatment regimes. Along the same line, an important advantage of artificial surfactants over animal-derived surfactants is that its composition can be modified in a rational manner, for example by adding esterase-resistant phospholipids or optimizing the peptide content, a possibility that should be explored in future studies.

In conclusion, we present a method for delipidation of rSP-C33Leu which together with production in *E*. *coli* followed by salt precipitation and resolubilization in organic solvents [25] allows efficient generation of large amounts of synthetic lung surfactant. A mixture of rSP-C33Leu and phospholipids has therapeutic effects that are comparable to poractant alfa in an animal model of neonatal RDS. This is an important step towards the development of novel surfactant-based treatment regimes for respiratory conditions in humans.

## Supporting information

S1 Fig SDS PAGE analysis of fractions from HPLC in Fig 3. Fraction 1 corresponds to the peak at 64% of 2-propanol and fraction 2 to the peak at 67% of 2-propanol in Fig 3.
(JPG)

S1 File Original SDS PAGE gels from Figs 2 and 3. (DOCX)

S2 File Original data from Fig 4A and 4B. (XLSX)

S3 File Original data from Fig 4C. (PDF)

S4 File Checklist. (PDF)
