# Supplementary figures and images for: Efficient delipidation of a recombinant lung surfactant lipopeptide analogue by liquid-gel chromatography
Source: PLoS One. 2019 Dec 4;14(12):e0226072. doi: 10.1371/journal.pone.0226072 (PMC6892477; doi:10.1371/journal.pone.0226072)

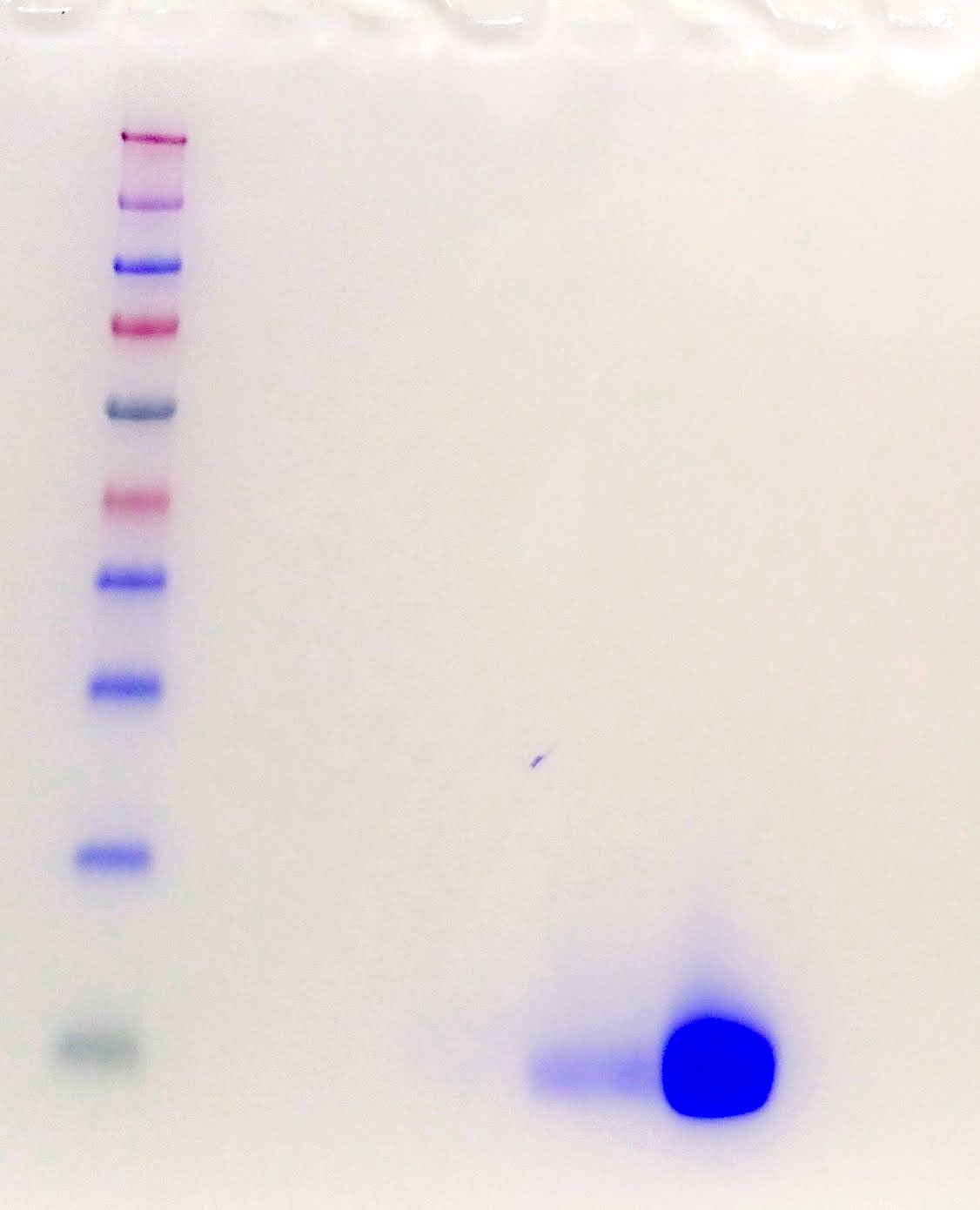

Supplement: S1 Fig — Fraction 1 corresponds to the peak at 64% of 2-propanol and fraction 2 to the peak at 67% of 2-propanol in Fig 3. (JPG) [file pone.0226072.s001.jpg]

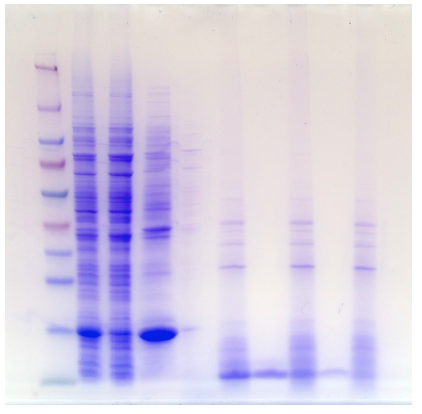


Original SDS-PAGE from Figure 1.


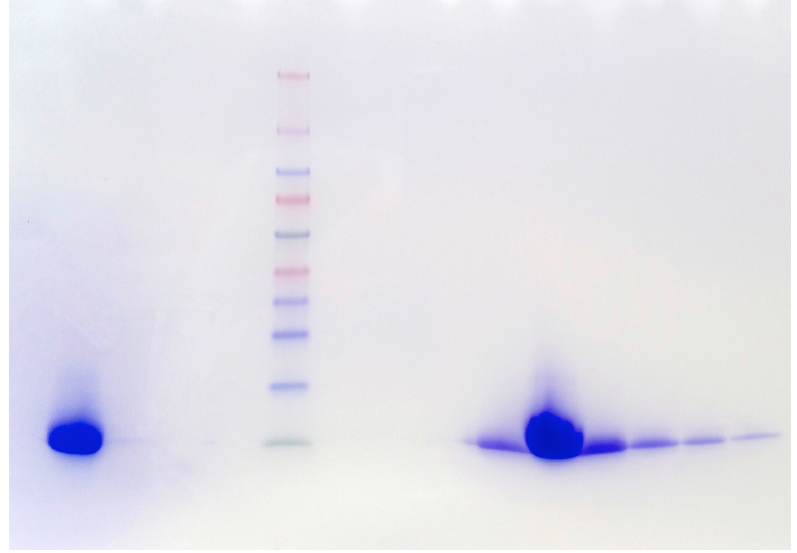


Original SDS-PAGE from Figure 2

Supplement: S1 File — (DOCX) [file pone.0226072.s002.docx]
