# Supplementary material for: Efficient delipidation of a recombinant lung surfactant lipopeptide analogue by liquid-gel chromatography
Source: PLoS One. 2019 Dec 4;14(12):e0226072. doi: 10.1371/journal.pone.0226072 (PMC6892477; doi:10.1371/journal.pone.0226072)

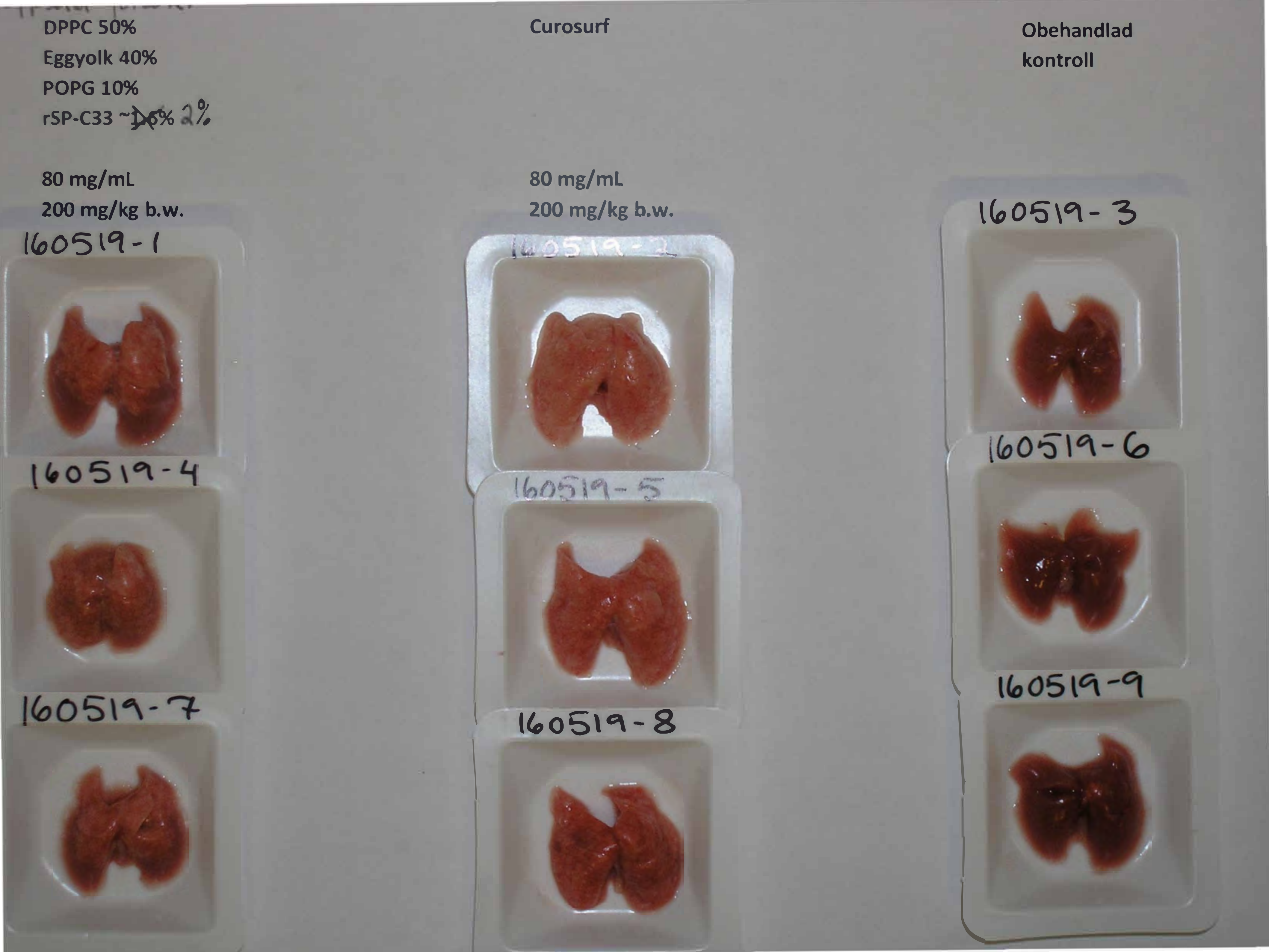

DPPC 50%
Eggyolk 40%
POPG 10%
rSP-C33 ~2%
80 mg/mL
200 mg/kg b.w.
Curosurf
80 mg/mL
200 mg/kg b.w.
Obehandlad
kontroll
160519-1
160519-2
160519-3
160519-4
160519-5
160519-6
160519-7
160519-8
160519-9

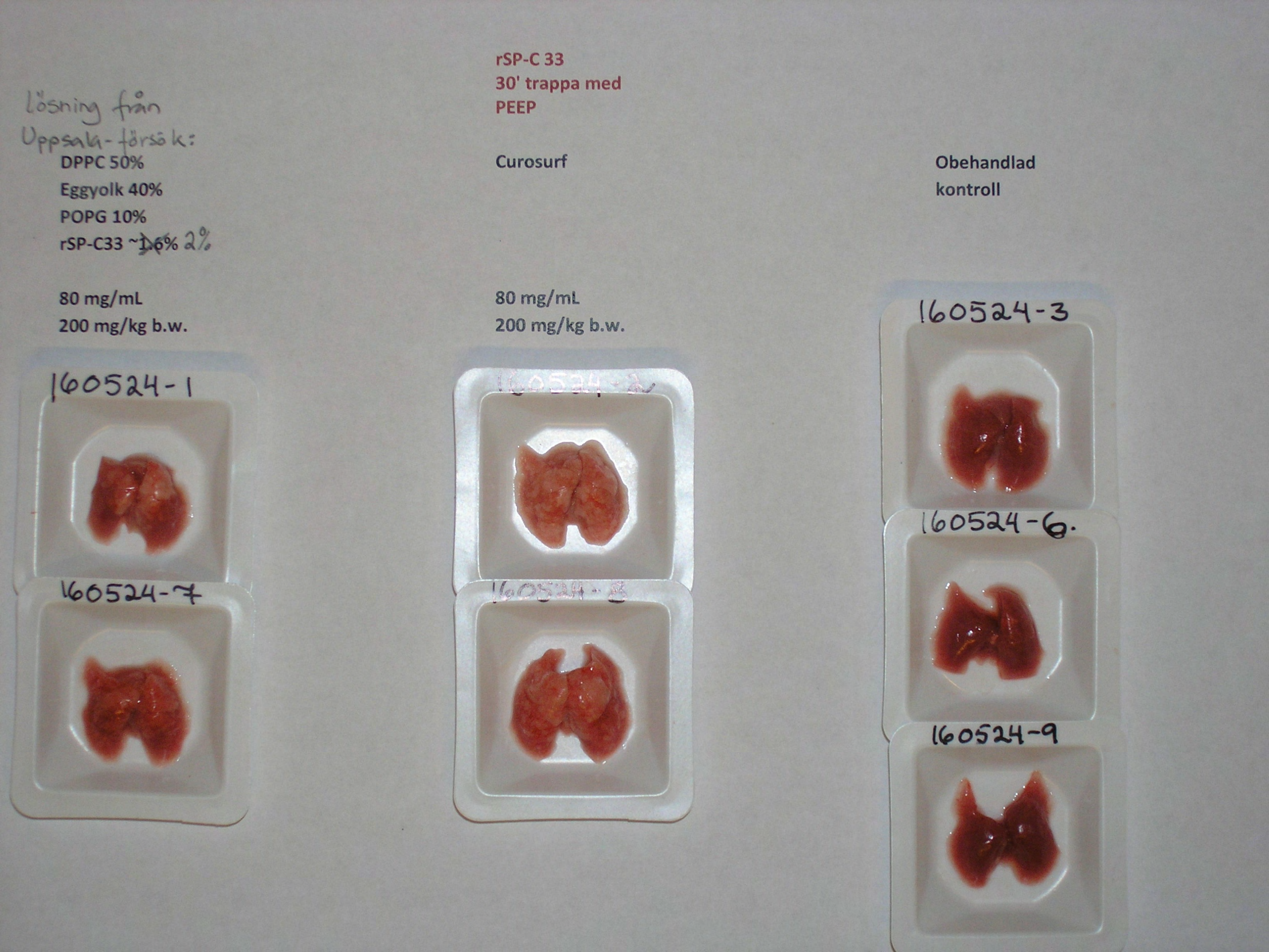
Lösning från Uppsala-försök:
DPPC 50%
Eggyolk 40%
POPG 10%
rSP-C33 ~~~1.6~~% 2%
80 mg/mL
200 mg/kg b.w.
160524-1
160524-7
rSP-C 33
30' trappa med
PEEP
Curosurf
80 mg/mL
200 mg/kg b.w.
160524-2
160524-8
Obehandlad
kontroll
160524-3
160524-6.
160524-9

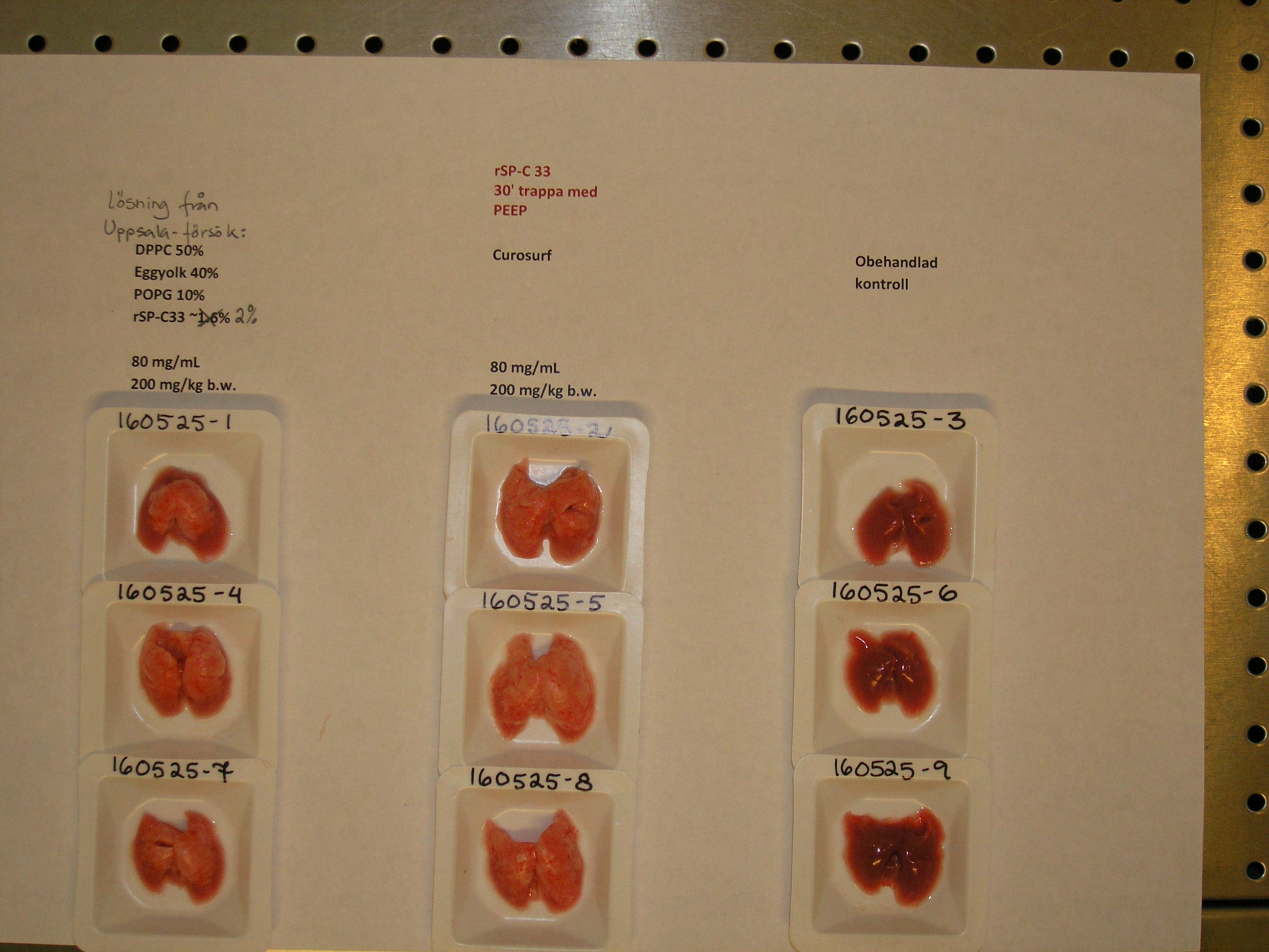
lösning från
Uppsala-försök:
DPPC 50%
Eggyolk 40%
POPG 10%
rSP-C33 ~1,6% 2%
80 mg/mL
200 mg/kg b.w.
rSP-C 33
30' trappa med
PEEP
Curosurf
80 mg/mL
200 mg/kg b.w.
Obehandlad
kontroll
160525-1
160525-2
160525-3
160525-4
160525-5
160525-6
160525-7
160525-8
160525-9

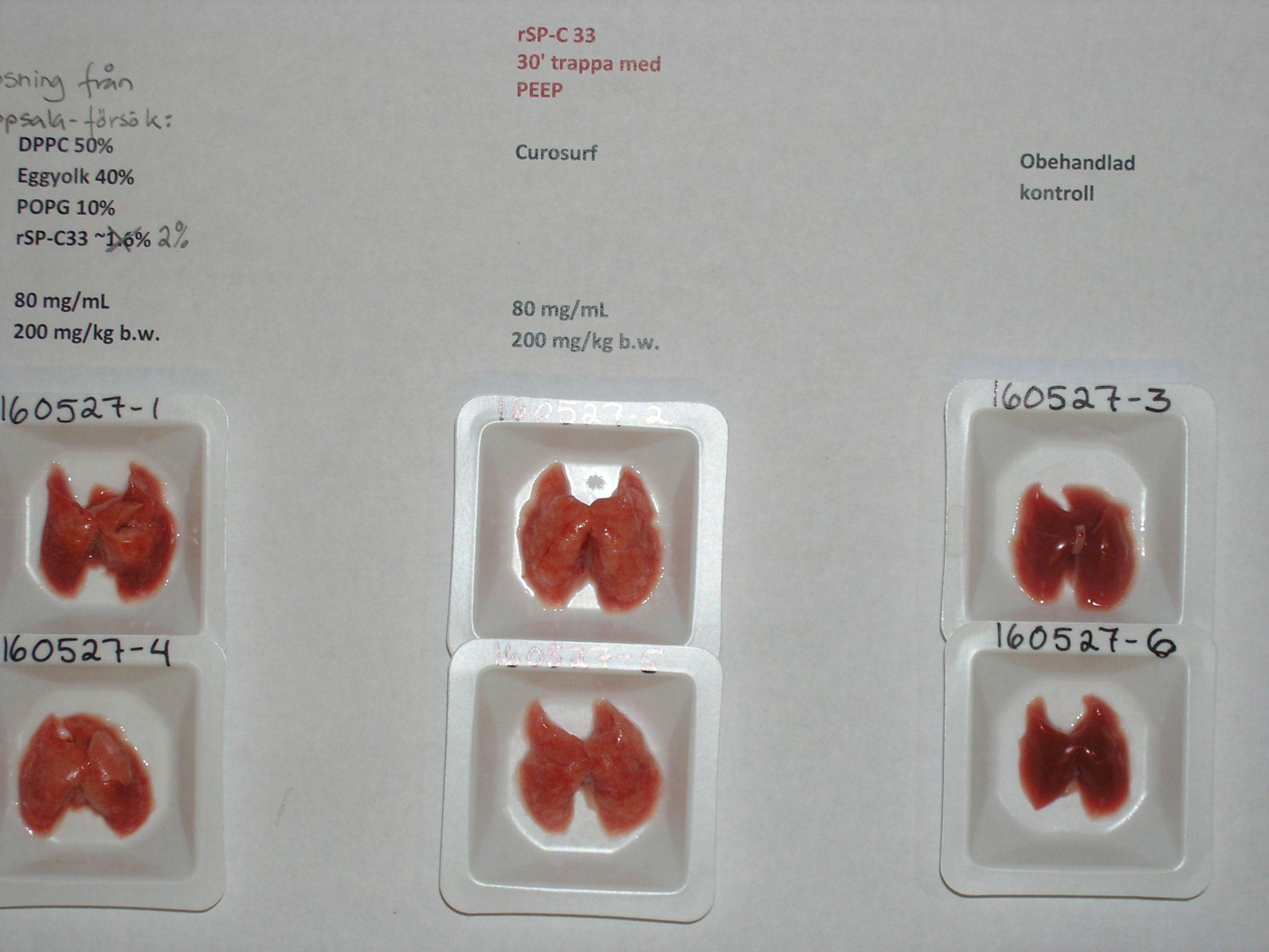
sning från
psala-försök:
DPPC 50%
Eggyolk 40%
POPG 10%
rSP-C33 ~1.6% 2%
80 mg/mL
200 mg/kg b.w.
rSP-C 33
30' trappa med
PEEP
Curosurf
80 mg/mL
200 mg/kg b.w.
Obehandlad
kontroll
160527-1
160527-2
160527-3
160527-4
160527-5
160527-6

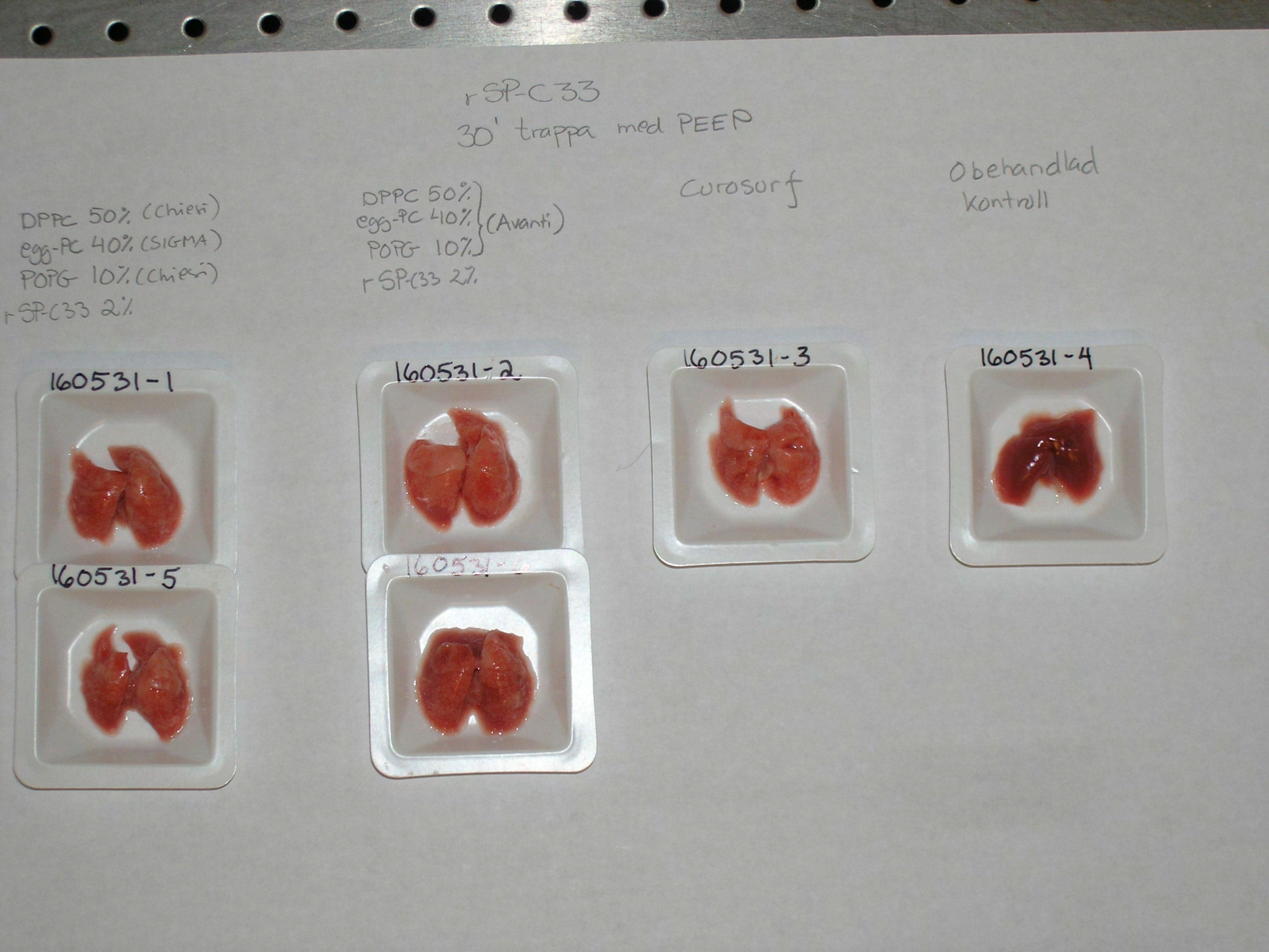
rSP-C33
30' trappa med PEEP
DPPC 50% (Chiesi)
egg-PC 40% (SIGMA)
POPG 10% (Chiesi)
rSP-C33 2%
DPPC 50%
egg-PC 40%
POPG 10%
(Avanti)
rSP-C33 2%
Curosurf
Obehandlad
kontroll
160531-1
160531-2
160531-3
160531-4
160531-5
160531-

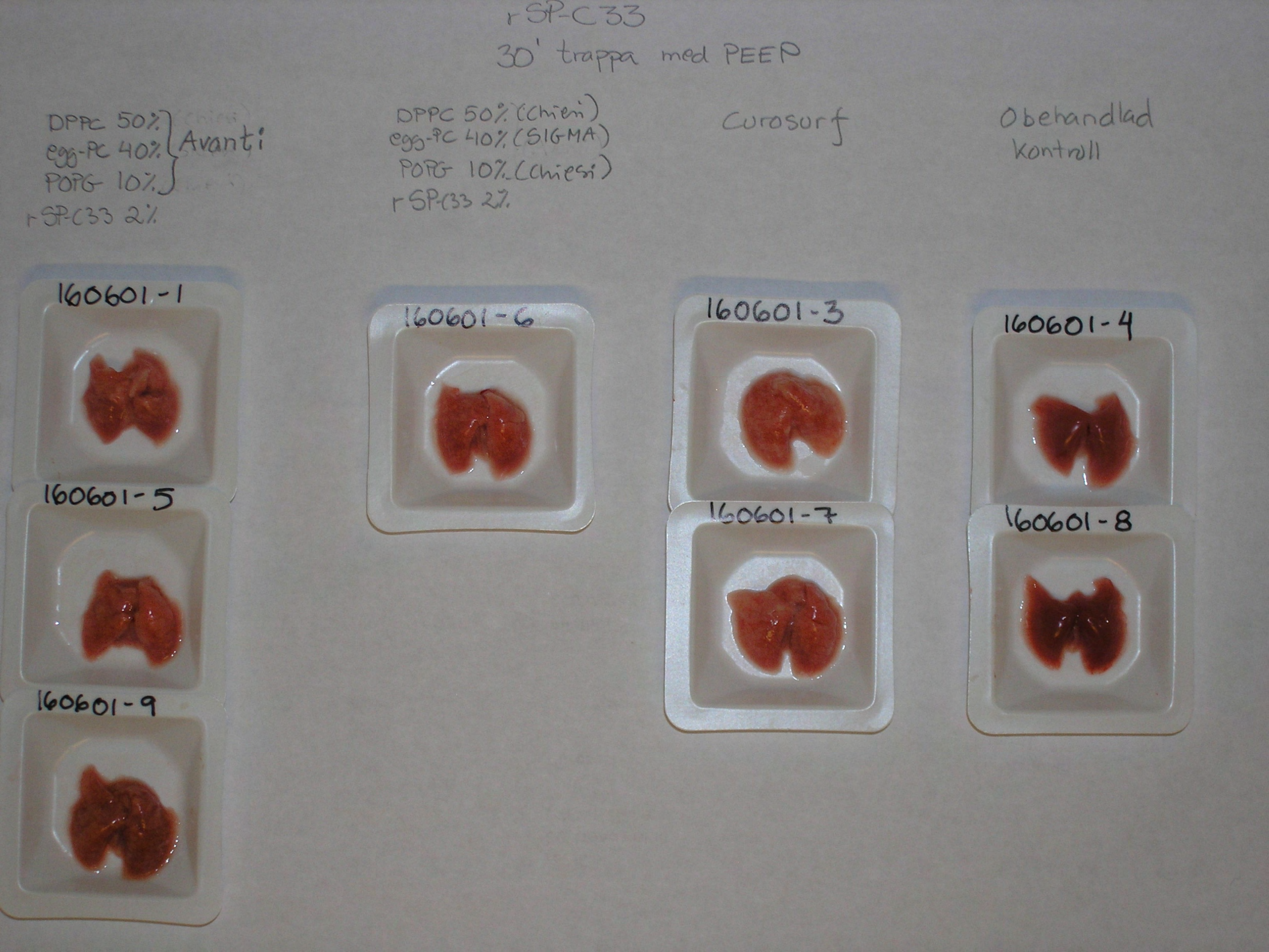
rSP-C33
30' trappa med PEEP
DPPC 50%
egg-PC 40%
POPG 10%
Avanti
rSP-C33 2%
DPPC 50% (Chiesi)
egg-PC 40% (SIGMA)
POPG 10% (Chiesi)
rSP-C33 2%
Curosurf
Obehandlad kontroll
160601-1
160601-5
160601-9
160601-6
160601-3
160601-7
160601-4
160601-8

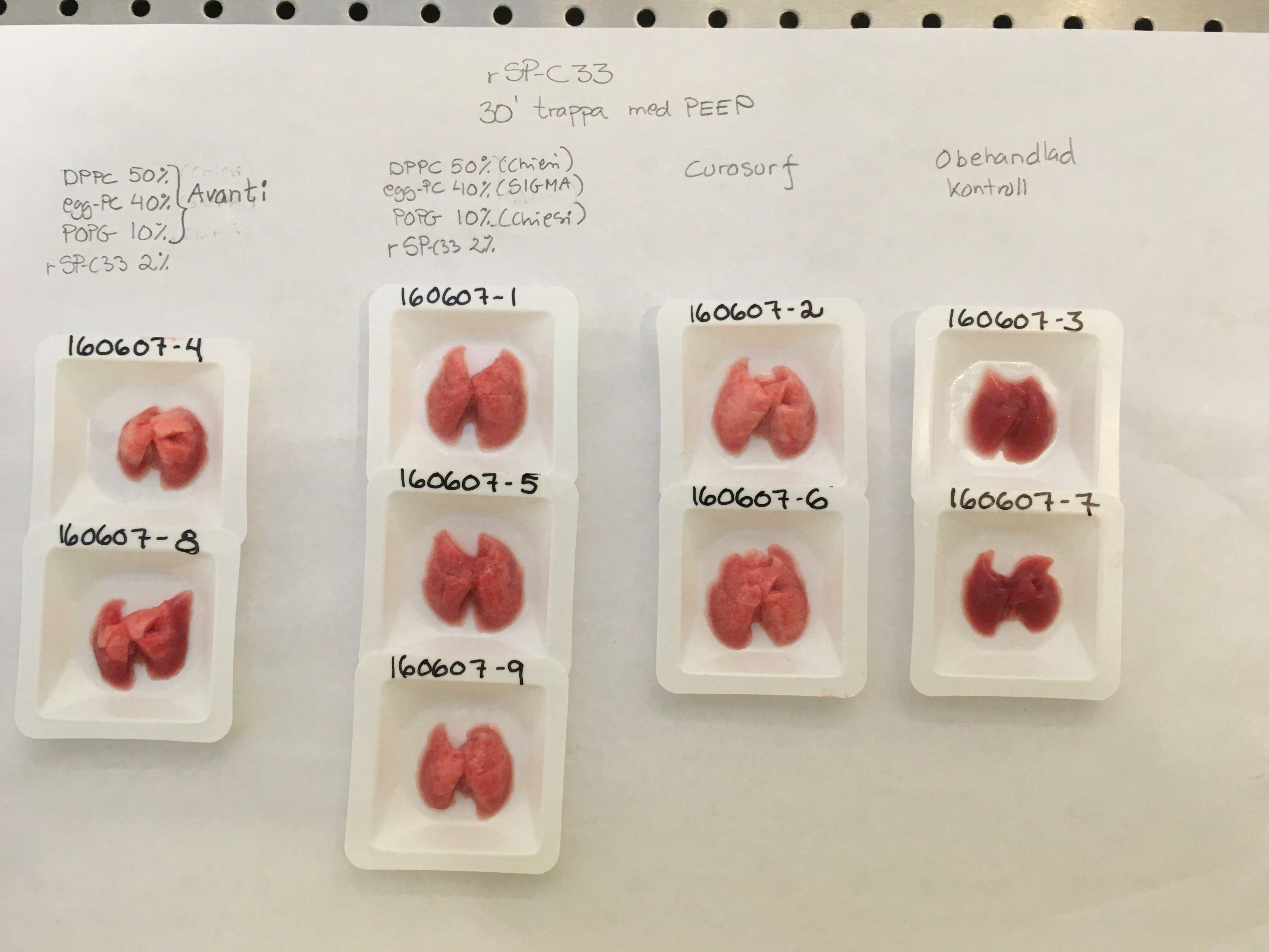
rSP-C33
30' trappa med PEEP
DPPC 50%
egg-PC 40%
POPG 10%
Avanti
rSP-C33 2%
DPPC 50% (Chiesi)
egg-PC 40% (SIGMA)
POPG 10% (Chiesi)
rSP-C33 2%
Curosurf
Obehandlad
kontroll
160607-4
160607-8
160607-1
160607-5
160607-9
160607-2
160607-6
160607-3
160607-7

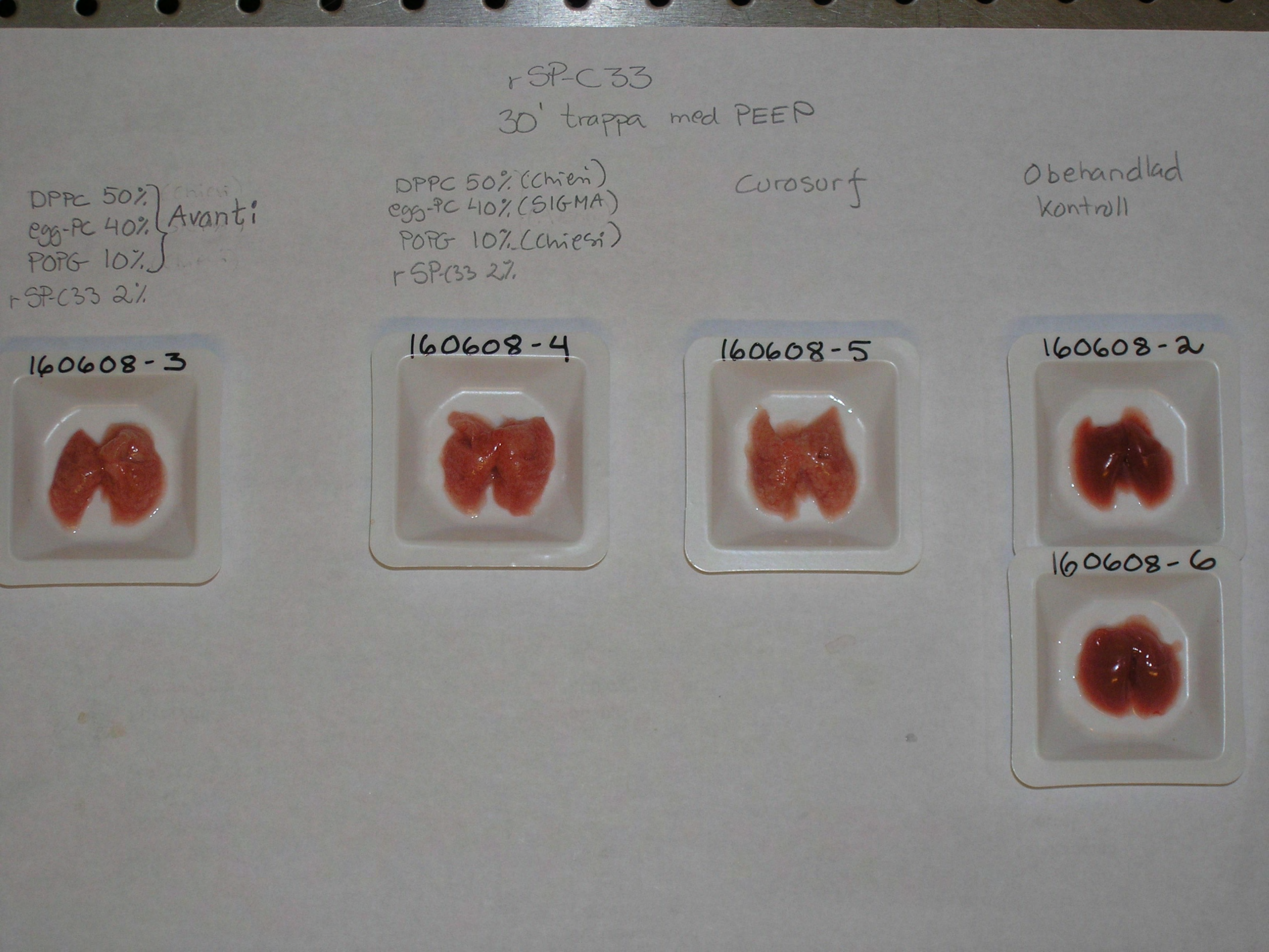
rSP-C33
30' trappa med PEEP
DPPC 50%
egg-PC 40%
POPG 10%
Avanti
rSP-C33 2%
DPPC 50% (Chiesi)
egg-PC 40% (SIGMA)
POPG 10% (Chiesi)
rSP-C33 2%
Curosurf
Obehandlad
kontroll
160608-3
160608-4
160608-5
160608-2
160608-6

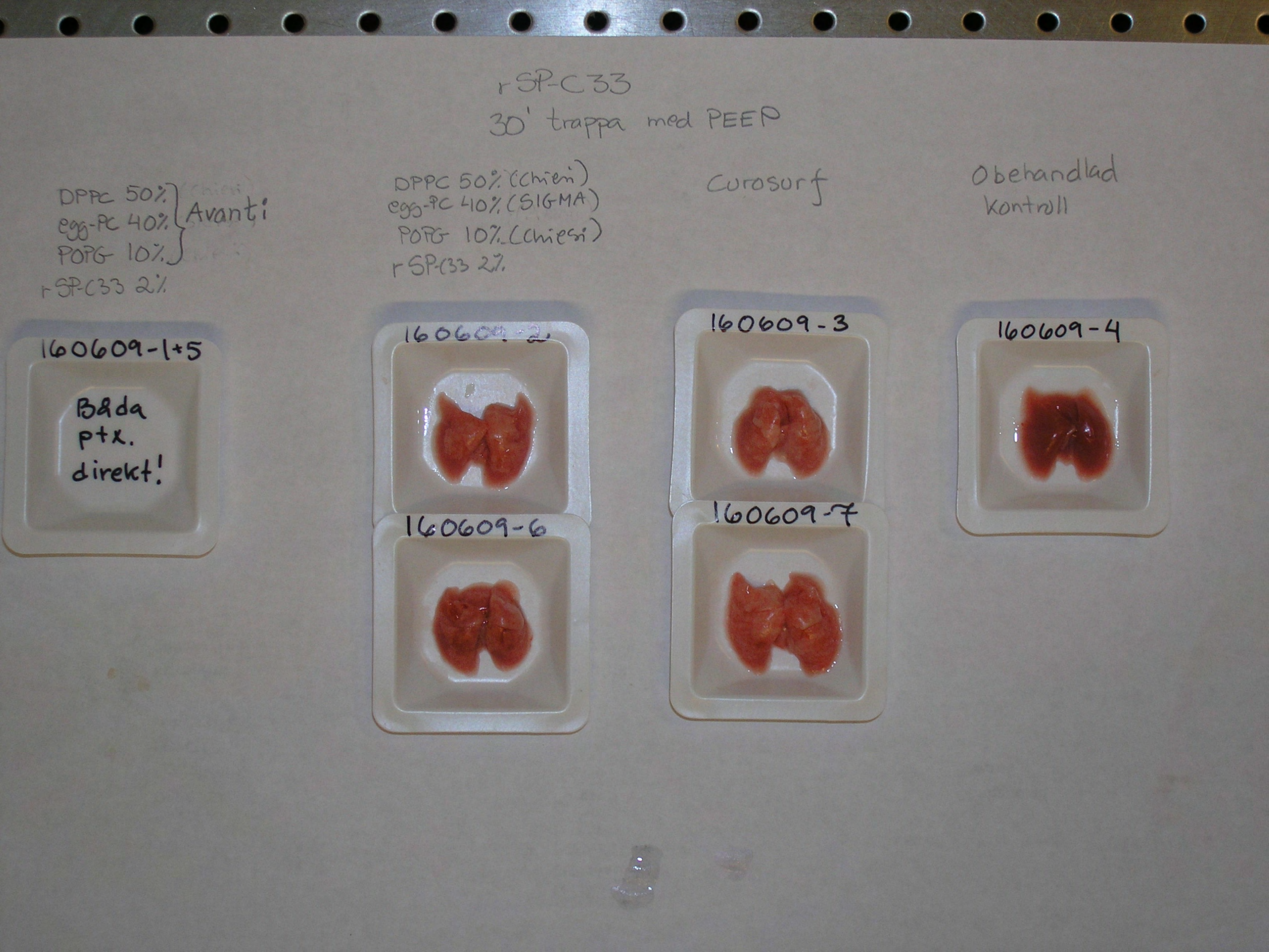
rSP-C33
30' trappa med PEEP
DPPC 50%
egg-PC 40%
POPG 10%
Avanti
rSP-C33 2%
DPPC 50% (Chiesi)
egg-PC 40% (SIGMA)
POPG 10% (Chiesi)
rSP-C33 2%
Curosurf
Obehandlad
kontroll
160609-1+5
Båda
ptx.
direkt!
160609-2
160609-6
160609-3
160609-7
160609-4

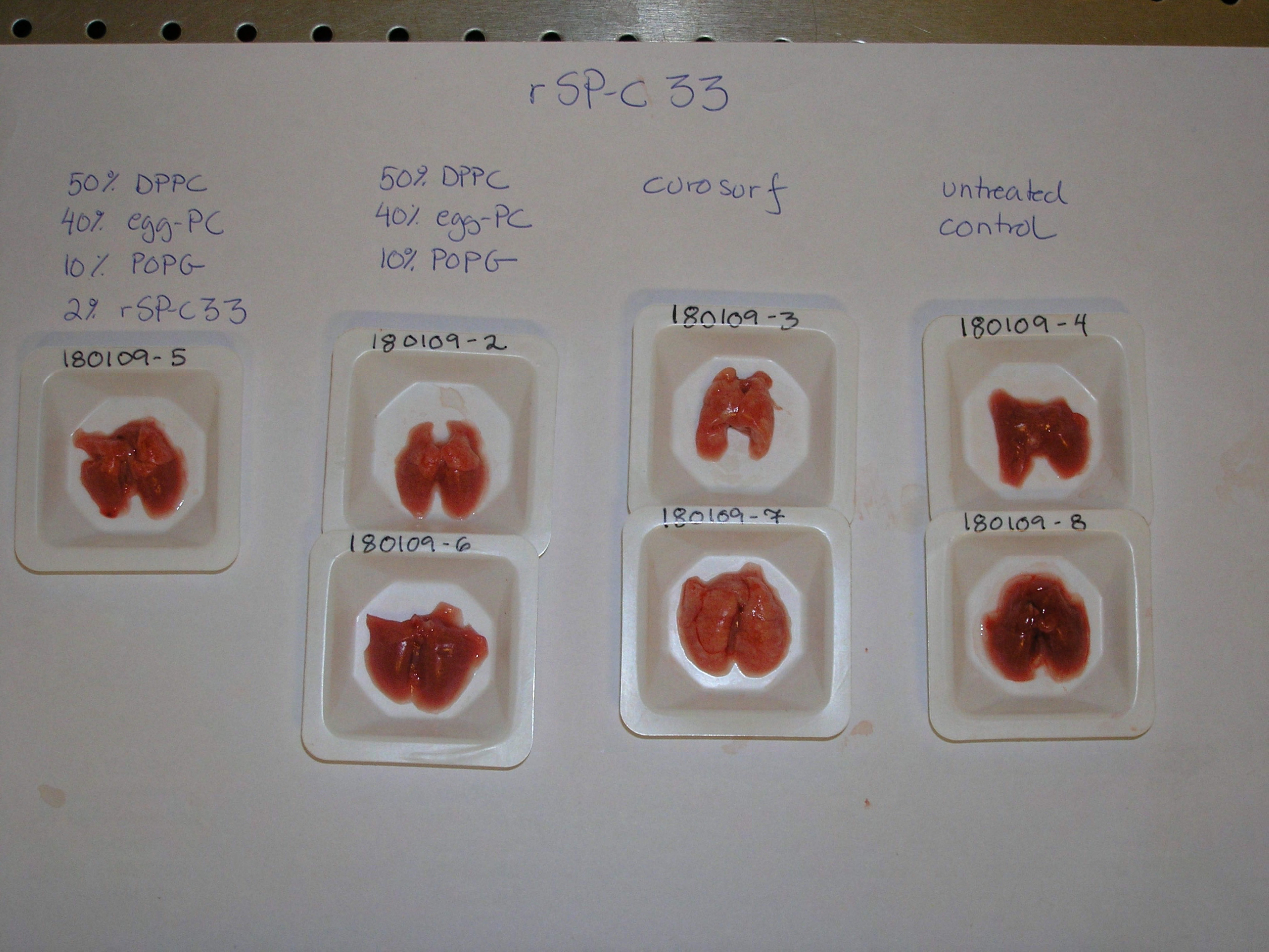
rSP-C 33
50% DPPC
40% egg-PC
10% POPG
2% rSP-C33
180109-5
50% DPPC
40% egg-PC
10% POPG
180109-2
180109-6
curosurf
180109-3
180109-7
untreated
control
180109-4
180109-8

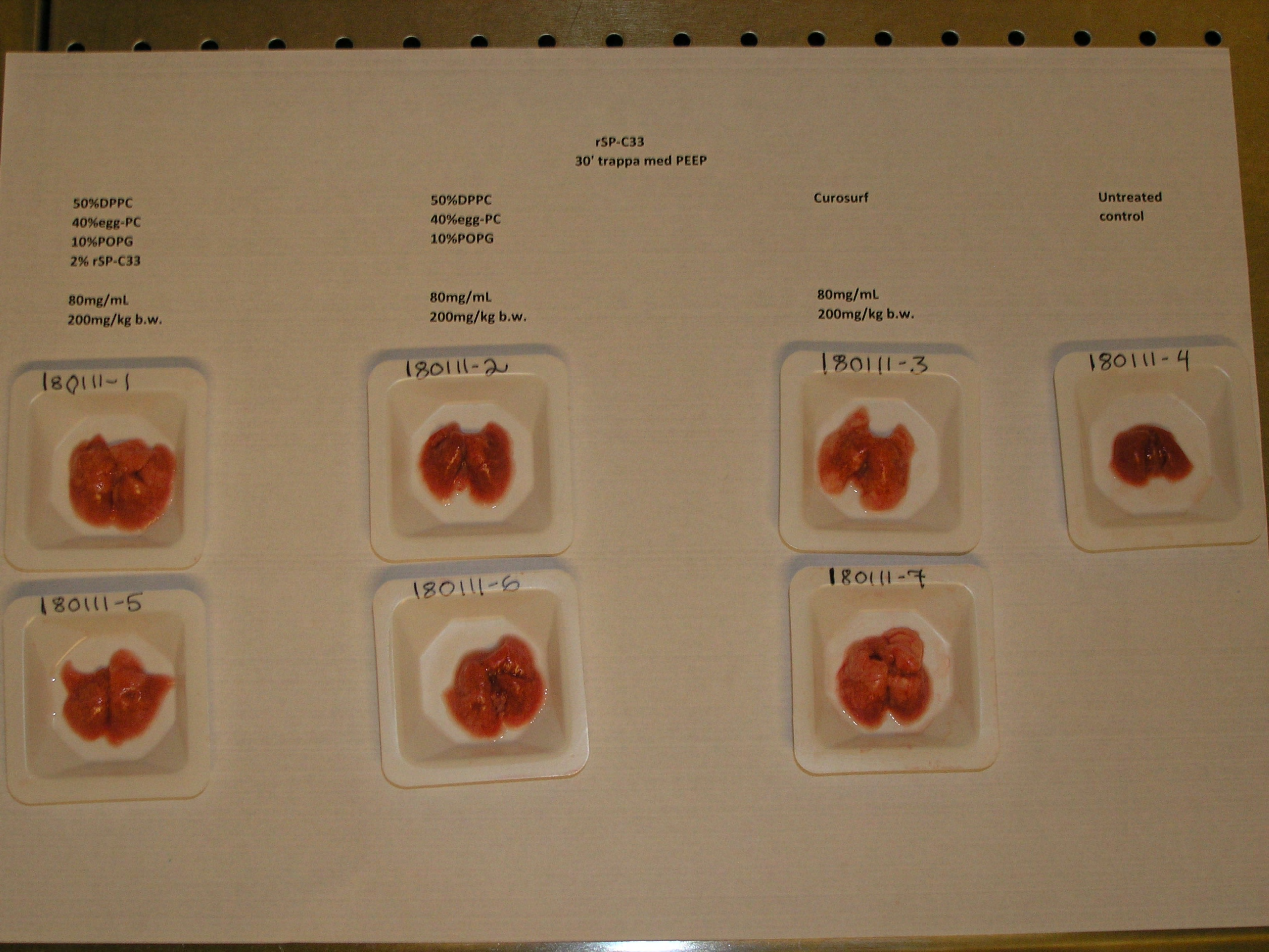
rSP-C33
30' trappa med PEEP
50%DPPC
40%egg-PC
10%POPG
2% rSP-C33
80mg/mL
200mg/kg b.w.
50%DPPC
40%egg-PC
10%POPG
80mg/mL
200mg/kg b.w.
Curosurf
80mg/mL
200mg/kg b.w.
Untreated
control
180111-1
180111-2
180111-3
180111-4
180111-5
180111-6
180111-7

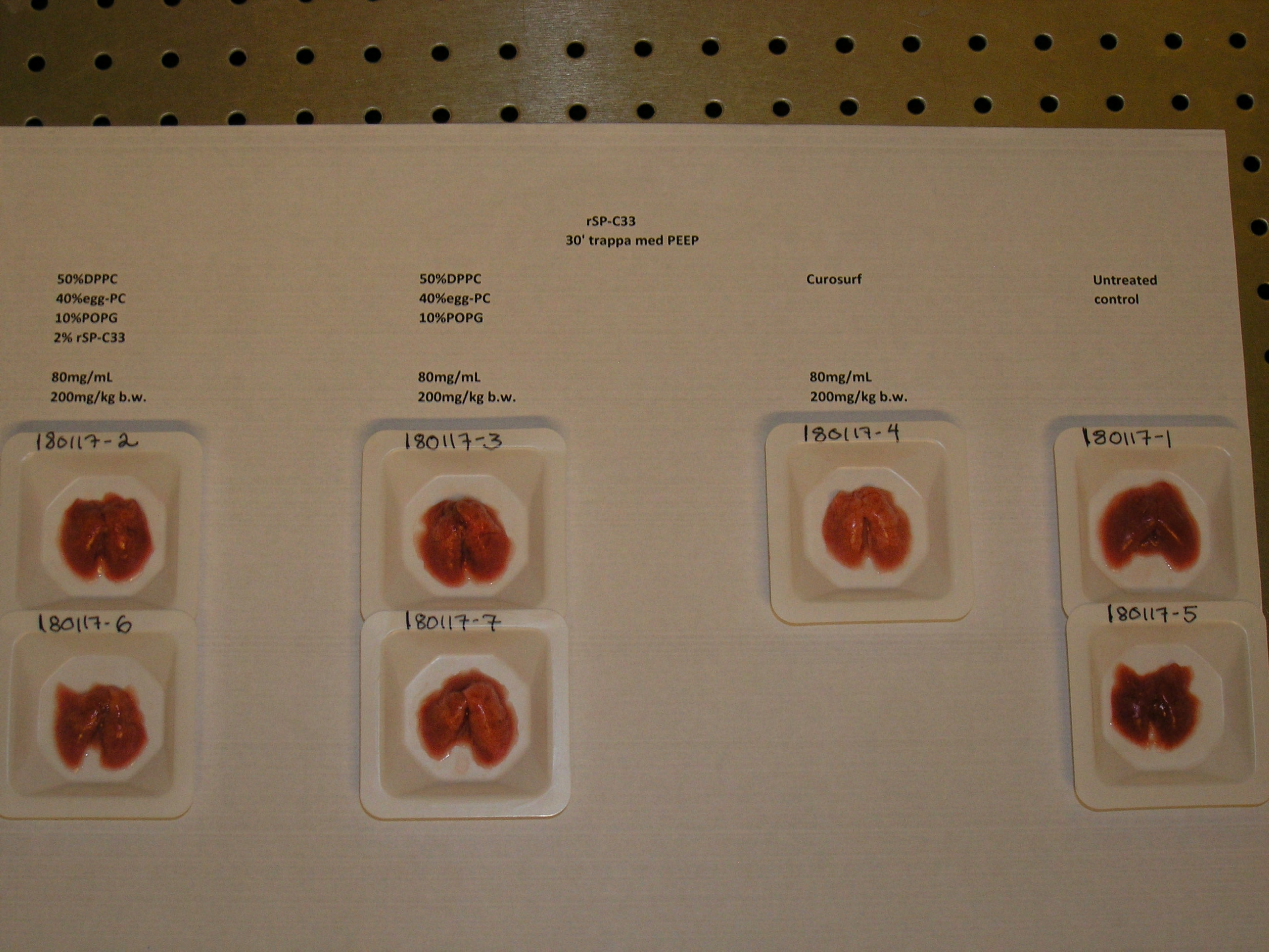
rSP-C33
30' trappa med PEEP
50%DPPC
40%egg-PC
10%POPG
2% rSP-C33
80mg/mL
200mg/kg b.w.
50%DPPC
40%egg-PC
10%POPG
80mg/mL
200mg/kg b.w.
Curosurf
80mg/mL
200mg/kg b.w.
Untreated
control
180117-2
180117-3
180117-4
180117-1
180117-6
180117-7
180117-5

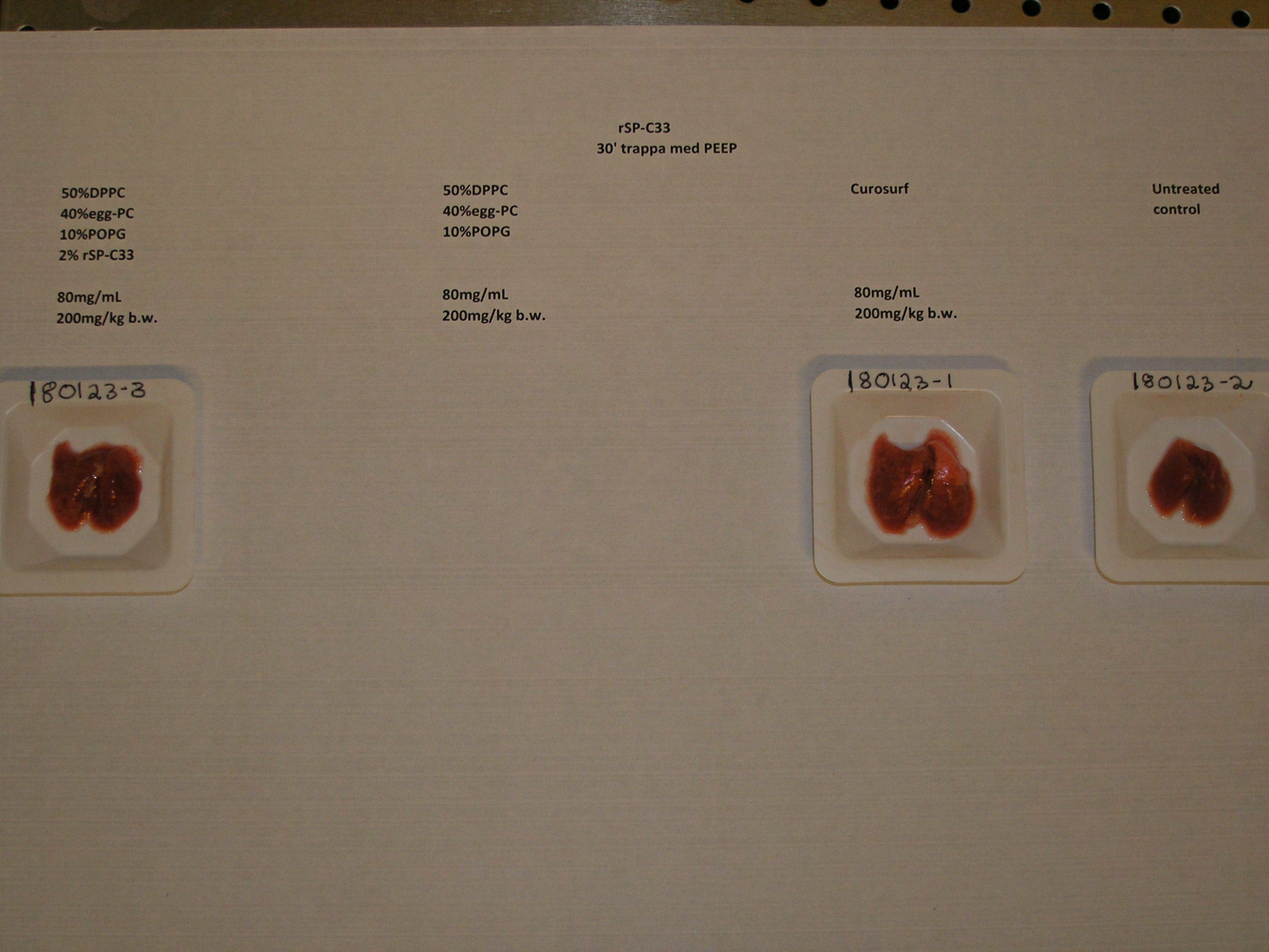
rSP-C33
30' trappa med PEEP
50%DPPC
40%egg-PC
10%POPG
2% rSP-C33
80mg/mL
200mg/kg b.w.
50%DPPC
40%egg-PC
10%POPG
80mg/mL
200mg/kg b.w.
Curosurf
80mg/mL
200mg/kg b.w.
Untreated
control
180123-3
180123-1
180123-2

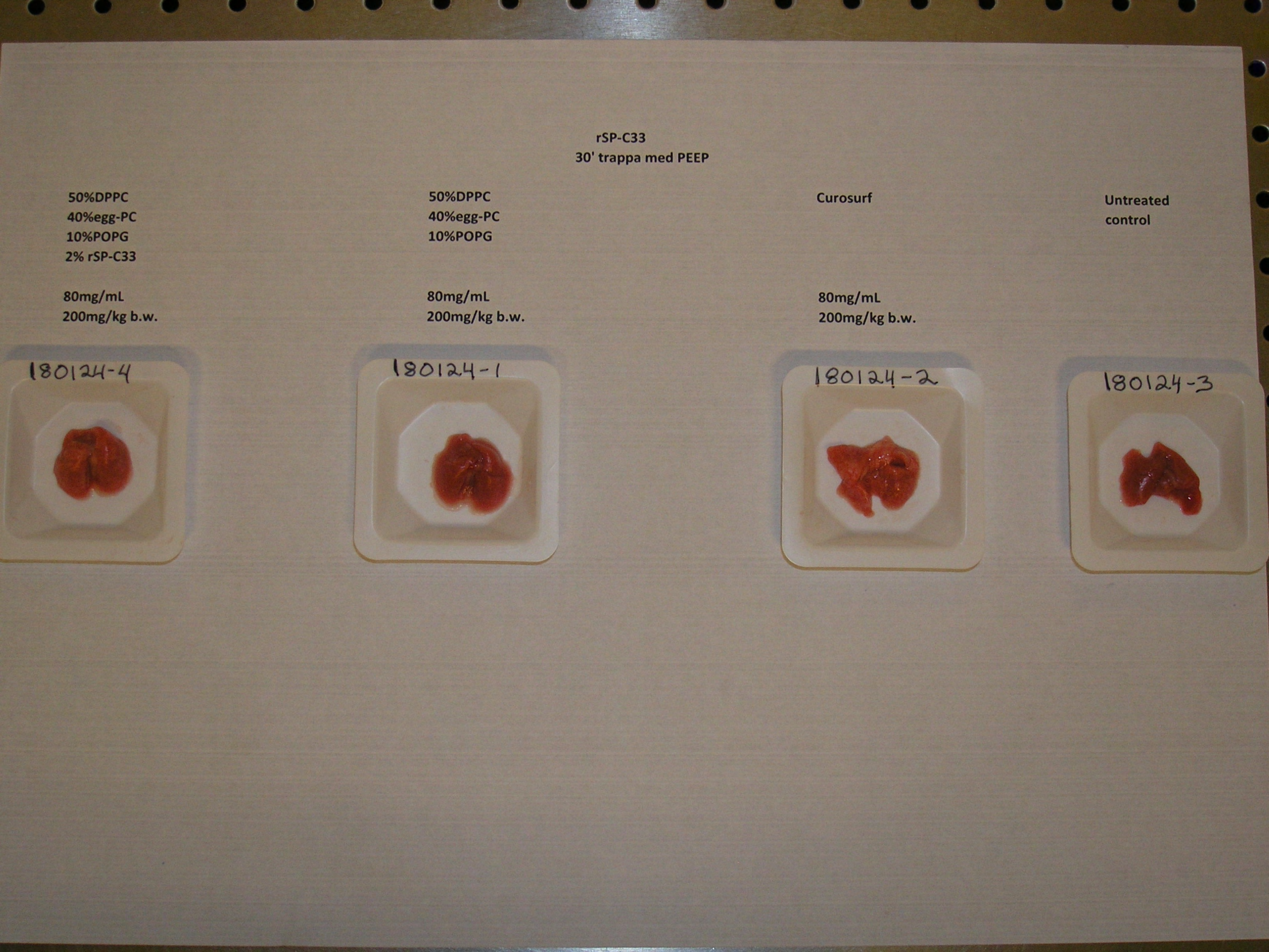
rSP-C33
30' trappa med PEEP
50%DPPC
40%egg-PC
10%POPG
2% rSP-C33
80mg/mL
200mg/kg b.w.
50%DPPC
40%egg-PC
10%POPG
80mg/mL
200mg/kg b.w.
Curosurf
80mg/mL
200mg/kg b.w.
Untreated
control
180124-4
180124-1
180124-2
180124-3

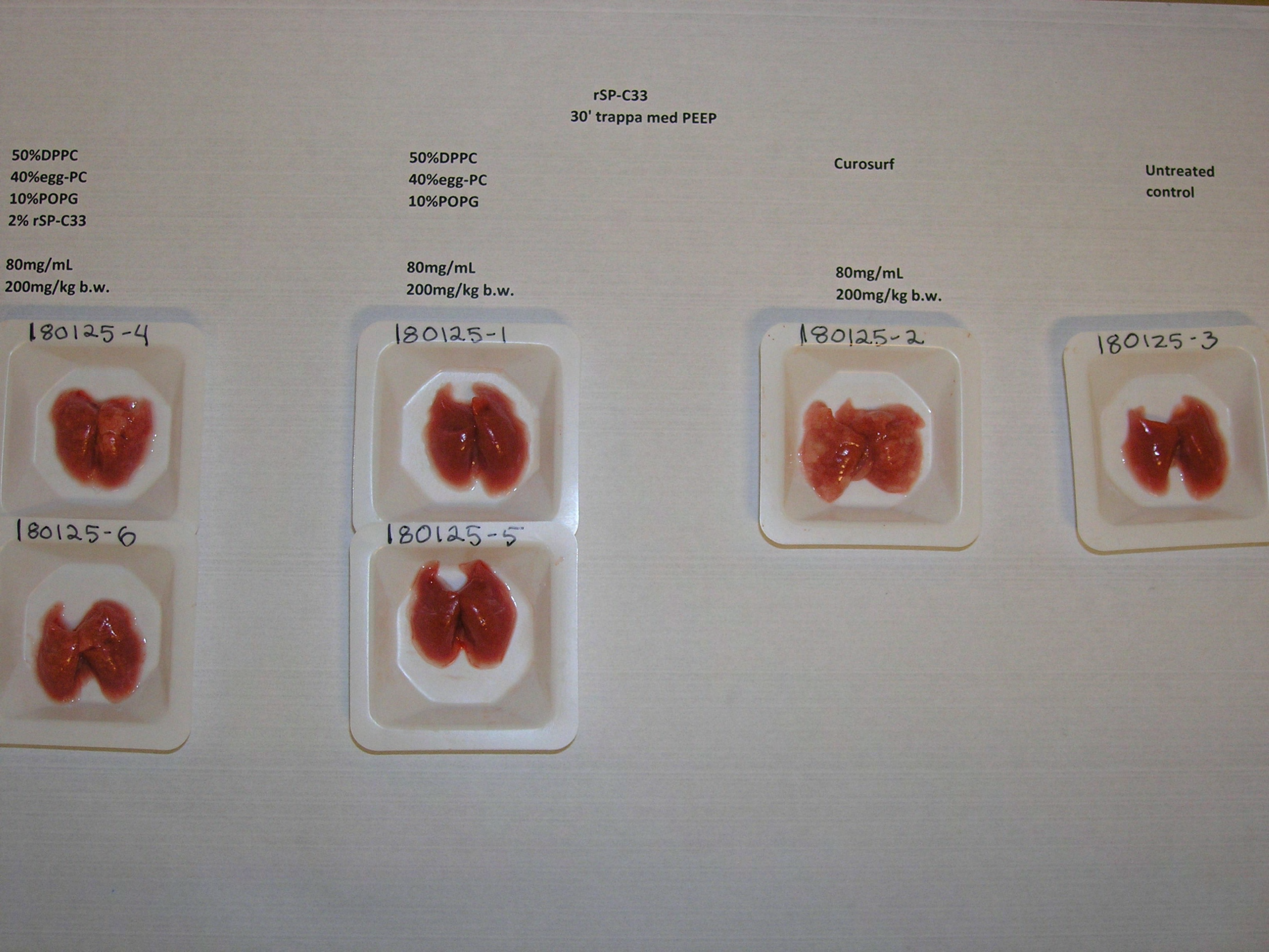
rSP-C33
30' trappa med PEEP
50%DPPC
40%egg-PC
10%POPG
2% rSP-C33
80mg/mL
200mg/kg b.w.
50%DPPC
40%egg-PC
10%POPG
80mg/mL
200mg/kg b.w.
Curosurf
80mg/mL
200mg/kg b.w.
Untreated
control
180125-4
180125-1
180125-2
180125-3
180125-6
180125-5

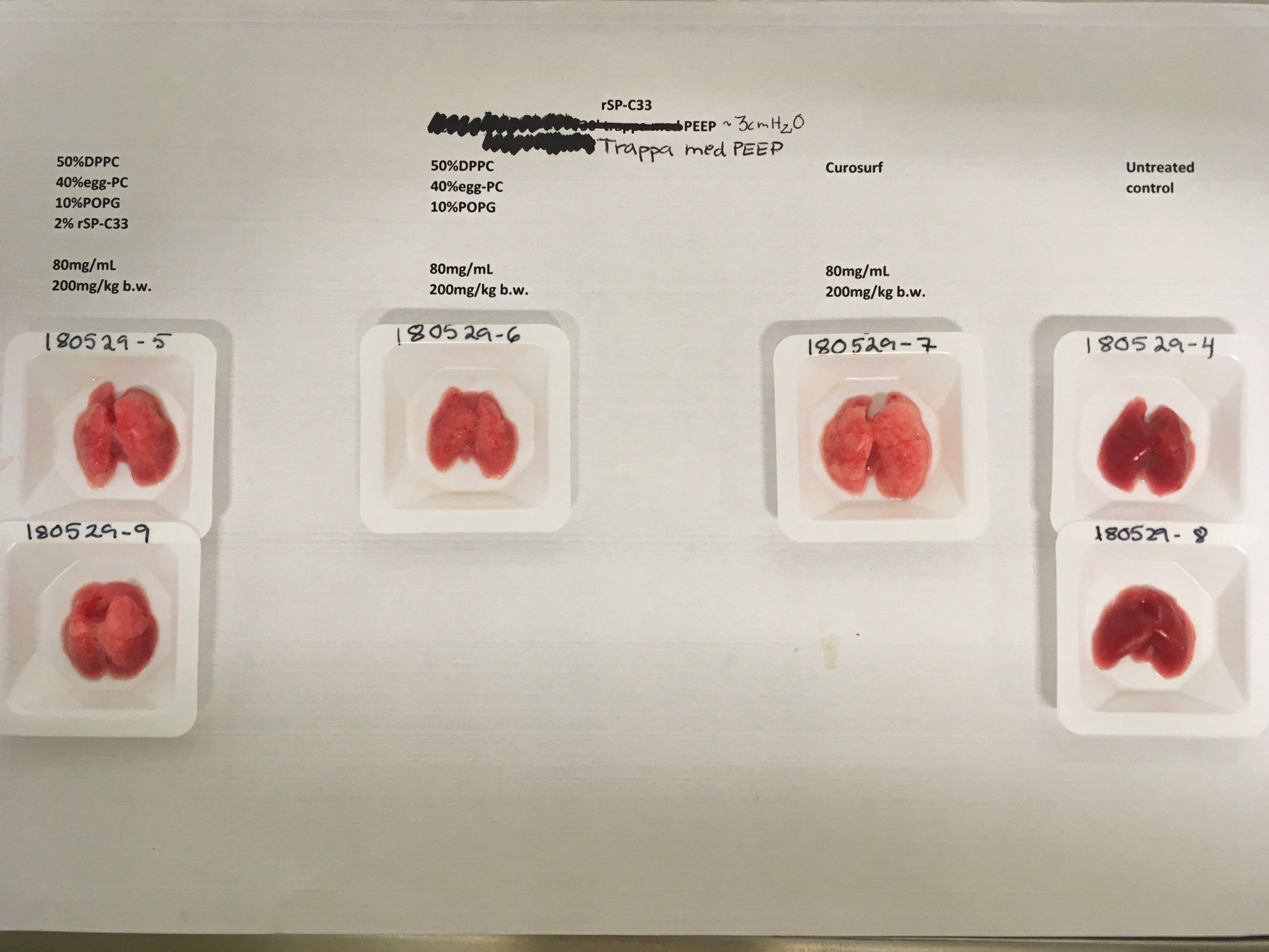
rSP-C33
[redacted] PEEP ~3cmH2O
[redacted] Trappa med PEEP
50%DPPC
40%egg-PC
10%POPG
2% rSP-C33
80mg/mL
200mg/kg b.w.
50%DPPC
40%egg-PC
10%POPG
80mg/mL
200mg/kg b.w.
Curosurf
80mg/mL
200mg/kg b.w.
Untreated
control
180529-5
180529-9
180529-6
180529-7
180529-4
180529-8

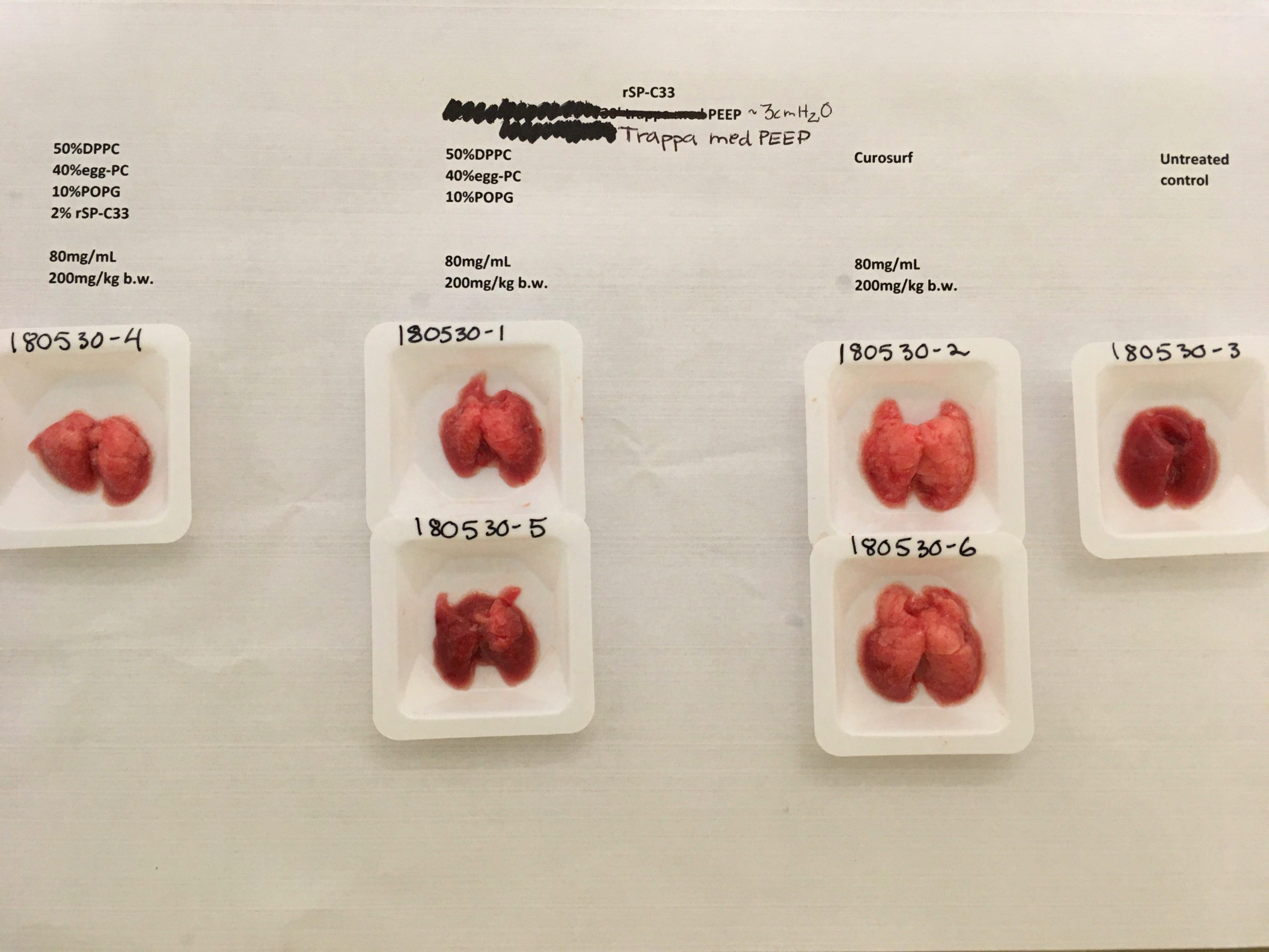

rSP-C33
[redacted] PEEP ~3cmH2O
[redacted] Trappa med PEEP
50%DPPC
40%egg-PC
10%POPG
2% rSP-C33
80mg/mL
200mg/kg b.w.
50%DPPC
40%egg-PC
10%POPG
80mg/mL
200mg/kg b.w.
Curosurf
80mg/mL
200mg/kg b.w.
Untreated
control
180530-4
180530-1
180530-5
180530-2
180530-6
180530-3

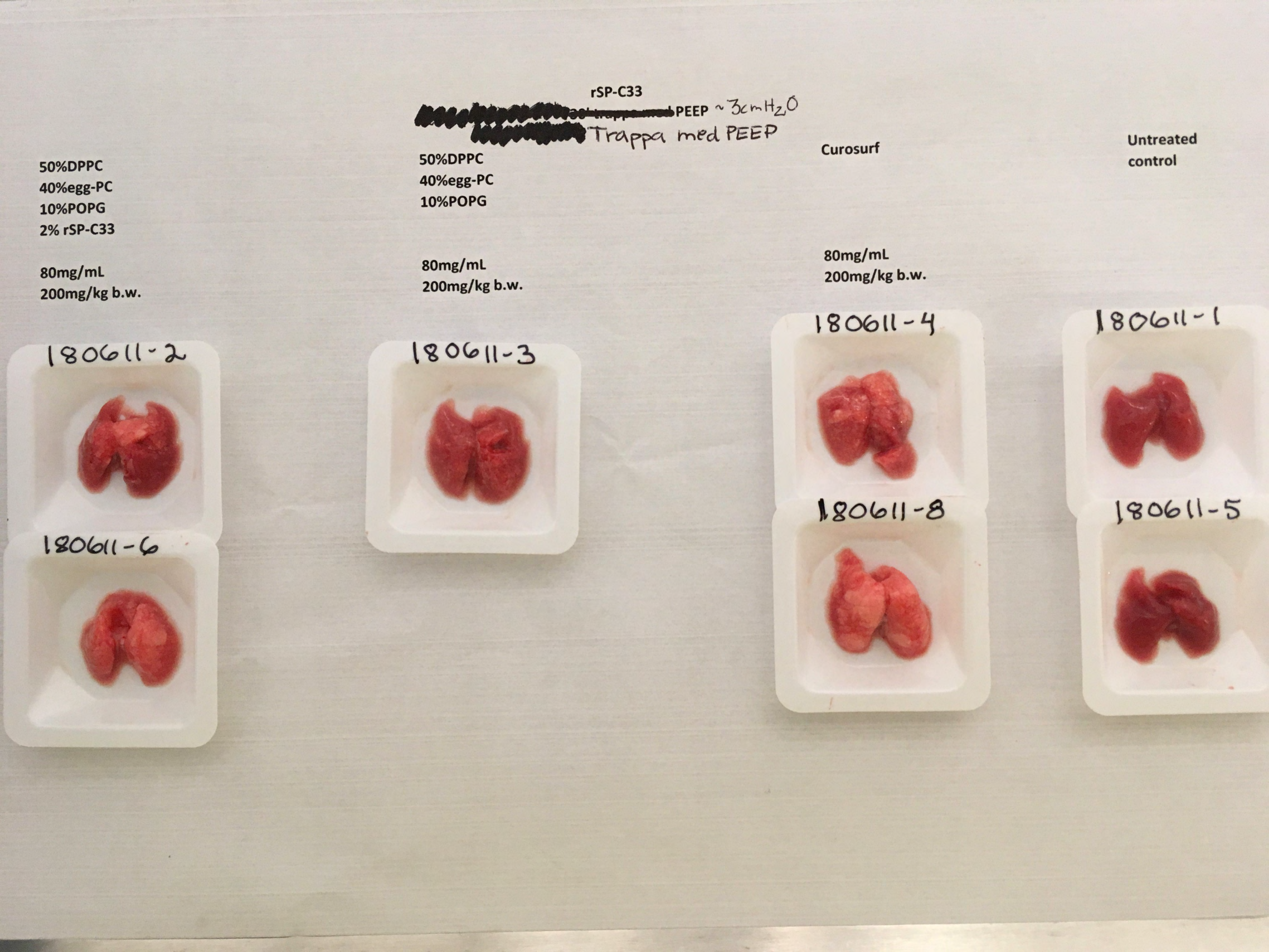
rSP-C33
[redacted] PEEP ~3cmH2O
[redacted] Trappa med PEEP
50%DPPC
40%egg-PC
10%POPG
2% rSP-C33
80mg/mL
200mg/kg b.w.
50%DPPC
40%egg-PC
10%POPG
80mg/mL
200mg/kg b.w.
Curosurf
80mg/mL
200mg/kg b.w.
Untreated
control
180611-2
180611-6
180611-3
180611-4
180611-8
180611-1
180611-5

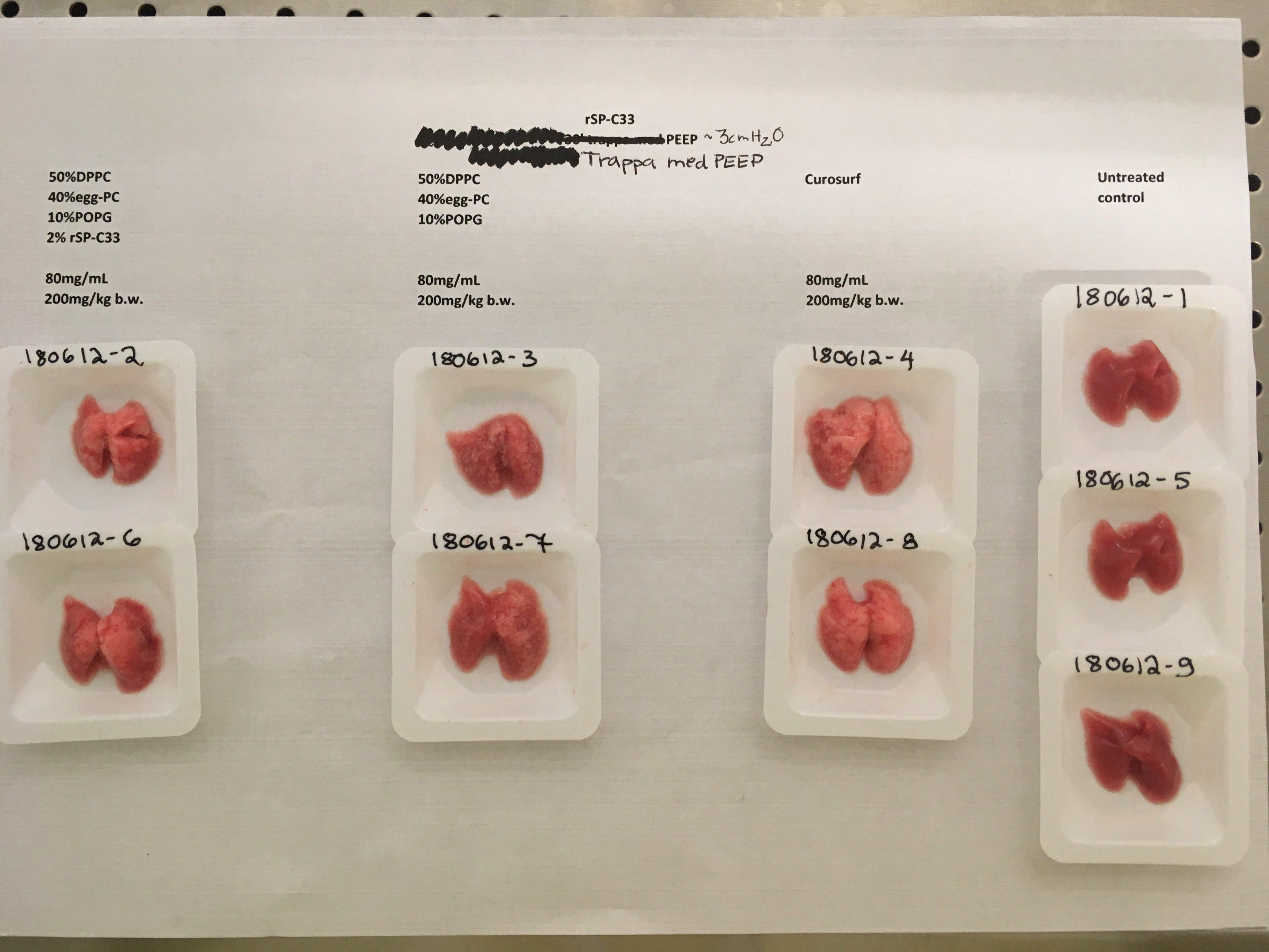
rSP-C33
[redacted] PEEP ~3cmH$_2$O
[redacted] Trappa med PEEP
50%DPPC
40%egg-PC
10%POPG
2% rSP-C33
80mg/mL
200mg/kg b.w.
50%DPPC
40%egg-PC
10%POPG
80mg/mL
200mg/kg b.w.
Curosurf
80mg/mL
200mg/kg b.w.
Untreated
control
180612-1
180612-2
180612-3
180612-4
180612-5
180612-6
180612-7
180612-8
180612-9

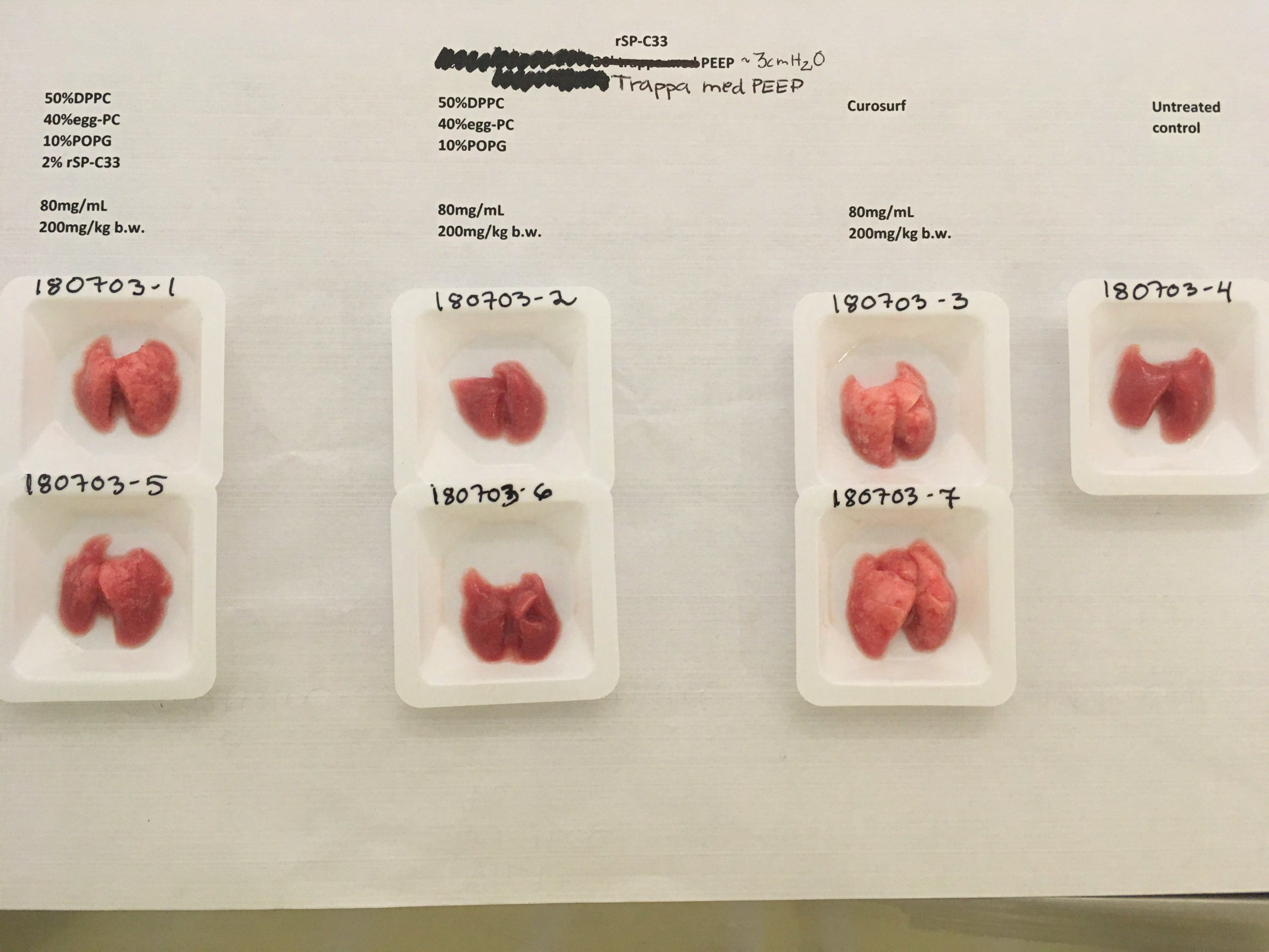

rSP-C33
[redacted] PEEP ~3cmH2O
[redacted] Trappa med PEEP
50%DPPC
40%egg-PC
10%POPG
2% rSP-C33
80mg/mL
200mg/kg b.w.
50%DPPC
40%egg-PC
10%POPG
80mg/mL
200mg/kg b.w.
Curosurf
80mg/mL
200mg/kg b.w.
Untreated
control
180703-1
180703-5
180703-2
180703-6
180703-3
180703-7
180703-4

rSP-C33

[redacted] PEEP ~3cmH_2O

[redacted] Trappa med PEEP

50%DPPC
40%egg-PC
10%POPG
2% rSP-C33

80mg/mL
200mg/kg b.w.

50%DPPC
40%egg-PC
10%POPG

80mg/mL
200mg/kg b.w.

Curosurf

80mg/mL
200mg/kg b.w.

Untreated control

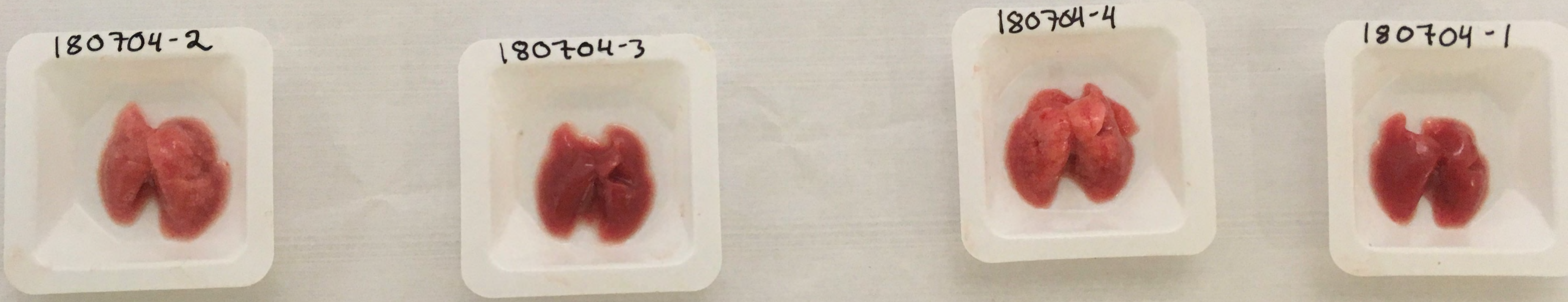

Supplement: S3 File — (PDF) [file pone.0226072.s004.pdf]
